# Neural stem cell-derived extracellular vesicles mitigate Alzheimer’s disease-like phenotypes in a preclinical mouse model

**DOI:** 10.1038/s41392-023-01436-1

**Published:** 2023-06-14

**Authors:** Ge Gao, Congcong Li, Yizhao Ma, Zhanping Liang, Yun Li, Xiangyu Li, Shengyang Fu, Yi Wang, Xiaohuan Xia, Jialin C. Zheng

**Affiliations:** 1 Center for Translational Neurodegeneration and Regenerative Therapy, Tongji Hospital affiliated to Tongji University School of Medicine, Shanghai, 200065 China; 2 Translational Research Center, Shanghai Yangzhi Rehabilitation Hospital affiliated to Tongji University School of Medicine, Shanghai, 201613 China; 3 Shanghai Frontiers Science Center of Nanocatalytic Medicine, Tongji University, Shanghai, 200331 China

**Keywords:** Neuroscience, Neural stem cells

**Dear Editor**,

Alzheimer’s disease (AD) is the most common neurodegenerative disorder and the No.1 cause of dementia in elderly with no effective treatments.^1^ The application of stem cell-derived extracellular vesicles (EVs) has emerged as a promising therapeutic strategy for AD.^2^ EVs are small bilipid layer-enclosed vesicles that display blood-brain barrier (BBB) penetrating ability and similar potency to their parental cells.^2^ The administration of EVs derived from neural stem cells (NSCs) and mesenchymal stem cell (MSCs) improved learning and memory functions of AD mice after EVs injection.^2^ NSC-derived EVs (NSC-EVs) have been reported to show better effects on improving neural recovery than MSC-derived EVs.^3^ However, due to ethical/religious concerns, supply constraints, limited expandability in culture, genetic/phenotypic instability issues, and potential immune responses of allogenic cell secretomes, NSCs may not be an excellent cell source for the production of clinical-grade EVs. Our previous studies obtained mouse fibroblast-derived induced NSCs (iNSCs) through somatic cell reprogramming, opening a new window for obtaining EVs derived from NSC-like cells.^4,5^ However, whether iNSC-derived (iNSC-EVs) can serve as promising substitutes of NSC-EVs in AD treatment remains unclear.

Here, we isolated EVs from the conditioned medium of mouse NSCs and iNSCs. iNSCs were characterized by immunocytochemistry (Supplementary Fig. 1). The purity of EVs was validated by western blotting, nano-particle tracking analysis, and electronic microscopy analyses (Supplementary Fig. 2). Five minutes after the intravenous administration of EVs labeled with a lipophilic carbocyanine fluorescent membrane labeling dye Dil, Dil signals were detected throughout mouse body (Supplementary Fig. 3) and in the brains (Supplementary Fig. 4) by in vivo fluorescence analyses. Immunohistochemical analyses detected Dil signals, colocalized with EV marker CD9, in Tuj1^+^ neurons, GFAP^+^ astrocytes, and Iba1^+^ microglia, in the cortex (Supplementary Fig. 5) and hippocampus (Supplementary Fig. 6), suggesting that EVs have been taken upby brain cells. Four-month-old APP/PS1 double transgenic mice that co-express five familial AD mutations (5 × FAD mice) were then intravenously administrated with either 200 μl EVs (0.5 μg/μl concentration) or an equal volume of PBS every three days for one month. After EVs treatment, 5 × FAD mice spent shorter times to find the platform in water maze versus PBS controls (Fig. 1a, b). EV treatment also increased distance traveled and time spent of 5 × FAD mice in the target quadrant in memory phase versus PBS controls (Fig. 1c), without affecting motor skills (Supplementary Fig. 7). Y maze test showed that EVs treatment significantly increased the numbers and ratios of novel arm entries of 5 × FAD mice versus PBS controls (Fig. 1d), without influencing motor performance of mice (Supplementary Fig. 8). EVs treatment also significantly increased the rearing numbers, decreased freezing time, and expanded central area entry numbers of mice in open field test versus PBS controls (Fig. 1e). Moreover, EVs treatment significantly reduced extracellular plaque density and average size in the prefrontal cortex (PFC) (Fig. 1f, g) and hippocampus (Supplementary Fig. 9) of 5 × FAD mice. Importantly, ELISA assay showed significantly decreased levels of Aβ_1-42_ in the PFC (Fig. 1h) and hippocampus (Supplementary Fig. 10) of EV-injected mice. Similarly, EVs treatment reduced phosphorylated Tau (pTau) propagation (Fig. 1i, j, Supplementary Fig. 11) and increased dendritic length and dendritic spine density (Fig. 1k-m, Supplementary Fig. 12) in the PFC and hippocampus of 5 × FAD mice, compared with PBS group. Moreover, EVs significantly improved the cognitive function of 5 × FAD mice even 1 month post administration as shown in water maze and fear conditioning tests (Supplementary Figs. 13, 14). Furthermore, Aβ_1-42_ accumulation (Supplementary Fig. 15) and pTau propagation (Supplementary Fig. 16) were dramatically reduced, and dendritic length and dendritic spine density (Supplementary Fig. 17) were significantly increased in the PFC and hippocampus of EV-injected mice, compared with PBS controls 1 month post EV administration, suggesting that the therapeutic effects of EVs has a great duration. Hence, iNSC-EVs showed comparable (no less if not more) therapeutic potentials than NSC-EVs on AD-like behavioral and pathological phenotypes.

**Fig. 1:**
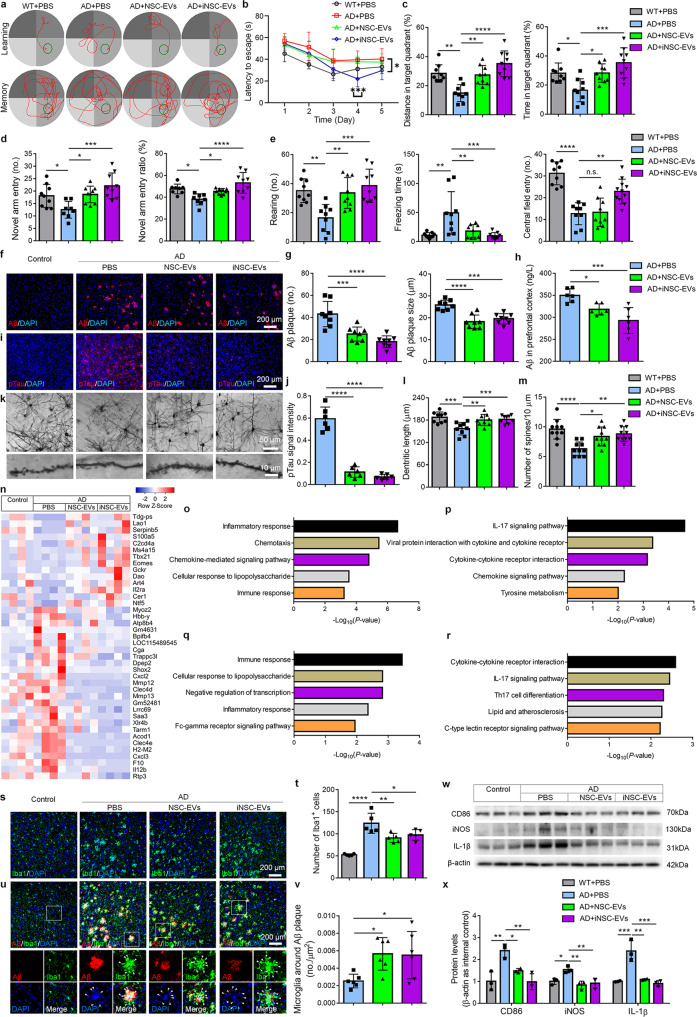
Intravenous administration of NSC-EVs and iNSC-EVs mitigate AD-like phenotypes in 5 × FAD mice. **a**–**d** Water maze: **a** Swim paths of mice while the platform was present (learning phase) or removed (memory phase); **b** Escape latencies in learning phase; **c** Platform crossing numbers and swimming distance in the target quadrant in memory phase (*n* = 9). **d** Y-maze: novel arm entry numbers and ratios (*n* = 9). **e** Open field test: rearing numbers, freezing times, and central field entry numbers (*n* = 9). **f** Aβ immunoreactivity in the PFC. **g** Aβ plaque density and average size were quantified using ImageJ (*n* = 8). **h** ELISA assay for the levels of Aβ_1-42_ in the PFC (*n* = 6). **i** pTau immunoreactivity in the PFC. **j** pTau signal intensity was quantified using ImageJ (*n* = 7). **k** Representative images of Golgi-stained PFC tissues (up) and high-power magnification images showing the somata and primary dendrites of neurons (down). **l, m** The dendritic length (**l**) and spine density (**m**) were quantified using ImageJ (*n* = 9~10). **n** Heatmap of top 40 DEGs among groups. **o**, **p** The top 5 GO terms (**o**) and KEGG pathways (**p**) of DEGs in comparison between NSC-EV- and PBS-injected mice. **q**, **r** The top 5 GO terms (**q**) and KEGG pathways (**r**) of DEGs in comparison between iNSC-EV- and PBS-injected mice. **s** Iba1 immunoreactivity in the PFC. (**t**) Numbers of Iba1^+^ cells were quantified using ImageJ (*n* = 5). **u** Iba1 and Aβ immunoreactivities in the PFC. **v** Numbers of Iba1^+^ cells around the surface of Aβ plaques were quantified using ImageJ (*n* = 6). **w**, **x** Representative blots (**w**) and quantification of CD86, IL-1β, and iNOS expression levels (**x**) in the PFC (*n* = 3). Scale bar: 200 μm (**j**, **s**, **u**). Error bars denote s.d. ns denotes no significance. *, **, ***, **** denote *p* < 0.05, *p* < 0.01, *p* < 0.001, *p* < 0.0001, respectively. The statistical difference among groups was assessed with the parametric one-way ANOVA with post-hoc Bonferroni test

We further collected EVs derived from MSCs, a widely used cell type in regenerative medicine, and fibroblasts, the origin cell of iNSCs (Supplementary Fig. 18). Water maze test showed greater platform crossing numbers and similar distance/time in target platform in iNSC-EV-injected mice, compared with MSC derived-EV (MSC-EVs)- and fibroblast-derived EV (FB-EV)-injected groups (Supplementary Fig. 19). Fear conditioning test suggested greater effects of iNSC-EVs on increasing freezing times of 5 × FAD mice, compared with MSC-EVs and FB-EVs (Supplementary Fig. 20). iNSC-EVs also exhibited greater inhibitory effects on Aβ_1-42_ accumulation (Supplementary Fig. 21) and pTau propagation (Supplementary Fig. 22), and better therapeutic effects on restoring the dendritic length and dendritic spine density (Supplementary Fig. 23) in the PFC and hippocampus of 5 × FAD mice versus MSC-EVs and FB-EVs. Therefore, iNSC-EVs showed greater potentials than MSC-EVs on alleviating AD-like phenotypes of 5 × FAD mice.

To understand the underlying mechanisms of iNSC-EV-mediated amelioration of AD-like phenotypes, we carried out RNA-seq analyses for RNAs isolated from PFC. The TOP 40 differentially expressed genes (DEGs) among groups revealed that AD-induced gene expression deregulation was restored by EVs administration (Fig. 1n). GO and KEGG analyses indicated a strong association of the DEGs in either NSC-EV- or iNSC-EV-injected groups versus PBS controls with inflammation-related terms and signaling pathways, respectively (Fig. 1o–r). Similar results were obtained *via* RNA-seq analyses for RNAs isolated from hippocampus, suggesting neuroinflammation, a key pathological feature and risk factors of AD,^1^ as a potential target of EVs treatment (Supplementary Fig. 24). RNA-seq results were corroborated by immunohistochemical analyses, showing that NSC-EVs and iNSC-EVs were mainly internalized by microglia, the resident immune cells in the brain (Supplementary Fig. 25). Moreover, EVs administration significantly reduced the numbers of Iba1^+^ cells and the degree of Iba1^+^ cell clustering around plaques in the PFC and hippocampus of 5 × FAD mice (Fig. 1s–v, Supplementary Fig. 26a–d). Western blotting results also indicated significant decline of the expression levels of pro-inflammatory proteins CD86, iNOS, and IL-1β in the PFC (Fig. 1w, x) and hippocampus (Supplementary Fig. 26e–h) of EV-injected mice versus PBS controls. In addition, astrocyte activation was also repressed by EVs, ascertained by reduced proportions of GFAP^+^ cells in both PFC and hippocampus of EV-injected 5 × FAD mice, compared with PBS-injected ones (Supplementary Fig. 27). To elucidate the potential mechanisms of EV-mediated immunomodulation, we analyzed the profiles of miRNAs, an important and enriched contents of EVs, in NSC-EVs and iNSC-EVs by microarray (Supplementary Fig. 28a). Microarray results indicated the enrichment of several miRNA families (e.g., let-7, miR-9, and miR-21) in both NSC-EVs and iNSC-EVs (Supplementary Fig. 28b–d), that are corroborated by qRT-PCR analyses (Supplementary Fig. 28e, f). Volcano plot further identified differentially expressed miRNAs in NSC-EVs (e.g., miR-34a, etc.) versus iNSC-EVs (e.g., miR-10b, etc.) (Supplementary Fig. 28b). Interestingly, around 40% of RNA-seq-identified down-regulated genes in EV-injected groups, including pro-inflammatory genes (e.g., *Il1b*, *Il12b*) and Aβ production-related ones (e.g., *Mmp13*, *Atp8b4*), are predicted to be direct targets of EV-enriched miRNAs (Supplementary Fig. 29). Knockdown of EV-enriched miRNAs including let-7i, miR-21a, and miR-10b significantly reduced inhibitory effects of EVs on Aβ-induced microglial activation (Supplementary Fig. 30), implicating miRNAs as key mediators of EV-dependent immunomodulation. Besides, immunohistochemistry results demonstrated a significant increase in the numbers of NeuN^+^ neurons (Supplementary Fig. 31), Ki67^+^ proliferating cells, and DCX^+^ neuronal precursors (Supplementary Fig. 32) in the PFC and hippocampus of EV-injected mice versus that of PBS controls, indicating comparable effects of iNSC-EVs and NSC-EVs on restoring neuroinflammation- and Aβ deposition-induced neuronal damage and neurogenesis impairment. Thus, our results suggest that anti-neuroinflammation be an important mechanism for EV-mediated amelioration of AD-like phenotypes.

In summary, we for the first time demonstrated that, after intravenous injection, iNSC-EVs exhibited comparable therapeutic effects with NSC-EVs on cognitive function, Aβ deposition, neuroinflammation, and neuroregeneration of 5 × FAD mice, suggesting iNSC-EVs as a promising succedaneum of NSC-EVs in AD treatment.

## Supplementary information

Supplementary materials

## Data Availability

All data needed to evaluate the conclusions in the paper are present in the paper or the Supplementary Materials. Any other raw data that support the findings of this study are available from the corresponding author upon reasonable request.
