# Supplementary material for: Neural stem cell-derived extracellular vesicles mitigate Alzheimer’s disease-like phenotypes in a preclinical mouse model
Source: Signal Transduct Target Ther. 2023 Jun 14;8:228. doi: 10.1038/s41392-023-01436-1 (PMC10264449; doi:10.1038/s41392-023-01436-1)
Supplement: Supplementary file 1 — Supplementary materials [file 41392_2023_1436_MOESM1_ESM.docx]

Supplementary Materials for

Neural stem cell-derived extracellular vesicles mitigate Alzheimer's disease-like phenotypes in a preclinical mouse model

Ge Gao^1ǂ^, Congcong Li^1ǂ^, Yizhao Ma^1ǂ^, Zhanping Liang^1ǂ^, Yun Li^1^, Xiangyu Li^1^, Shengyang Fu^1^, Yi Wang^2,3*^, Xiaohuan Xia^1,3*^, Jialin C. Zheng^1,3*^

^1^Center for Translational Neurodegeneration and Regenerative Therapy, Tongji Hospital affiliated to Tongji University School of Medicine, Shanghai 200065, China. ^2^Translational Research Center, Shanghai Yangzhi Rehabilitation Hospital affiliated to Tongji University School of Medicine, Shanghai 201613, China. ^3^Shanghai Frontiers Science Center of Nanocatalytic Medicine, Tongji University, Shanghai 200331, China.

Correspondence to: [windyiwang@foxmail.com](mailto:windyiwang@foxmail.com); [xiaohuan_xia1@163.com](mailto:xiaohuan_xia1@163.com); [jialinzheng@tongji.edu.cn](mailto:jialinzheng@tongji.edu.cn).

**This PDF file includes:**

Materials and Methods

Figures. S1 to S32

**Materials and Methods**

Mice

5×FAD and C57BL/6 mice were purchased from Shanghai Model Organisms Center, Inc. All mice were housed in isolated ventilated cages (maximum five mice per cage) in the Comparative Medicine animal facilities of Tongji University School of Medicine (YUSM). All mice were maintained on a 12/12-hours light/dark cycle, 24~26 °C, 40~70% humidity with sterile pellet food and water *ad libitum*. Cages were checked daily to ensure animal welfare. All procedures were conducted according to protocols approved by the Institutional Animal Care and Use Committee (IACUC) of TUSM (reference number: SYXK (HU) 2014-0026).

Isolation of mouse NSCs

NSCs were isolated from C57BL/6 mouse fetal brains. Briefly, NSCs were obtained from embryonic day 13.5 mouse cortical tissues and cultured with NSC culture medium containing NeuroCult® NSC Basal Medium (Stem Cell Technologies), NeuroCult® NSC Proliferation Supplements (Stem Cell Technologies), 20 ng/mL FGF2 (BioWalkersville), 20 ng/mL EGF (BioWalkersville) and 2 μg/mL heparin (Sigma), N2 supplement (Gibco), 2 mM L-glutamine (ThermoFisher), 100 U/ml penicillin & streptomycin (ThermoFisher) to form neurospheres. Primary neurospheres were then dissociated into single cells and re-plated for next round of neurosphere formation. NSCs were purified after three rounds of neurosphere formation.

Isolation of mouse microglia

Microglia were isolated from C57BL/6 mouse fetal brains. Briefly, postnatal day 1 mouse brains were dissected out and digested at 37 °C for 30 min in 0.25% trypsin-EDTA (Gibco) supplemented with 0.05% DNase I (Roche). Digestion was stopped by FBS (Invitrogen). Tissue sediment was centrifuged at 1500 rpm for 5 min at 4 °C. After trituration, dissociated cells were cultured in DMEM (Gibco) supplemented with 10 ng/mL GM-CSF and 10% FBS, 50 U penicillin and 50 mg/mL streptomycin at 37 °C. Culture dishes were coated with 100 mg/mL Poly-D-Lysine (Sigma) and 5 mg/mL Fibronectin (Sigma). The culture medium was replaced every 3 days. Mouse primary microglia in the glial cell-mixed cultures were induced to detach by shaking. Floating microglia were collected by 1500 rpm centrifugation for 5 min at 4 °C.

Reprogramming of mouse fibroblast into iNSCs

Mouse fibroblasts were derived from mouse embryos at embryonic day 14. Briefly, all the internal organs, head and spinal cord were removed from embryos. The remaining skin tissues were washed twice with PBS, and dissociated with 0.25% trypsin-EDTA solution. Mouse fibroblasts were cultured in high glucose medium supplemented with 10% FBS, 1% non-essential amino acid (non-AA), 100 U/ml penicillin, 100 μg/ml streptomycin at 37 °C in a 5% CO2 humidified atmosphere.

Mouse fibroblasts were directly reprogramed into iNSCs. Briefly, mouse fibroblasts were incubated in the mixed virus-containing supernatants overnight. 10 μg/mL polybrene (Millipore) was added to facilitate virus transfection. Infected fibroblasts were cultured in NSC culture medium 1 day after the second infection. NSC culture medium was replaced every two days. Day 28 after retroviral transduction, colonies were formed, manually picked and suspended into single cells to generate neurospheres. Floating primary neurospheres were collected after culturing for 4-6 days and re-plated into Poly-D-Lysine/Fibronectin-coated 6-well plates. Cells were collected after reaching 80% confluency and re-suspended into single cells for a second round of neurosphere formation. After 3 rounds of selection and enrichment, cells were collected for iNSC characterization.

Aβ oligomer preparation and Aβ-induced microglial activation in vitro

Aβ_42_ peptide solution (1 mM) was prepared by dissolving Aβ_42_ peptide (GL Biochem, Shanghai) in 221.7 μl HFIP (1,1,1,3,3,3-hexafluoro-2-propanol) (Sigma-Aldrich). The solution was placed at room temperature (RT) for 1 h, and then kept on ice for 10 min. Afterwards, the solution was aliquoted into non-siliconized microcentrifuge tubes (100 μl solution containing 0.45 mg Aβ_42_), and dried overnight at RT. The residues were dissolved in 20 μl dimethyl sulfoxide (DMSO), added with F12 medium to make a 100 μM stock solution, incubated at 4 °C overnight, and then centrifuged at 14,000×g for 10 min at 4 °C. The Aβ oligomers were presented in the supernatant, that was confirmed by immunoblot using anti-Aβ antibody (1:1000, BioLegend).

To mimic Aβ-induced microglial activation *in vivo*, primary microglia were treated with Aβ oligomer (dose: 10 μM) for 48 h at 37 °C. The activation of microglia was confirmed by examining the expression levels of transcripts corresponding to pro-inflammatory genes (*Tnf* and Il1b) and anti-inflammatory genes (*CD206* and *Ym1*) in microglia, and that of pro-inflammatory cytokines (TNF-α and IL-1β) in the conditioned medium.

Agonist/antagonist/siRNA and transfection

The antagomiR control, antagomiR-9, antagomiR-21a, antagomiR-34a, antagomiR-10b, and antagomir-let7i were purchased from GenePharma (GenePharma). Transfection of 20 nM antagomiR-9, antagomiR-21a, antagomiR-34a, antagomiR-10b, antagomir-let7i, or their corresponding controls was performed using the Lipofectamine 2000 reagent (Invitrogen) according to the manufacturer’s instruction.

Isolation of EVs

EVs were isolated from conditioned medium of NSCs and iNSCs culture. Briefly, conditioned media was first centrifuged at 300 g for 10 min to remove cells, at 3,000 g for 20 min to remove cellular debris, at 10,000 g for 30 min to remove intracellular organelles. The supernatant was filtered using a 0.22 μm syringe filter, and then centrifuged at 100,000 g for 4 h to collect EVs. Centrifugation was carried out at 4 °C. EVs were resuspended in PBS and stored in -80 °C for future use.

Staining of EVs with Dil

EV solution was gently mixed with Dil in PBS (1:1000) and incubated for 30 min in the dark at RT. The mixture was washed with PBS once, filtered with 0.22 μm filter, and ultracentrifuged at 100,000 g for 2 h at 4 °C, leaving any unbound dye to be discarded. Mice were given intravenous administration of Dil-labeled EVs or PBS. After 5 min, mice were sacrificed for brain tissues. Brain tissues were sectioned to brain slices. After sectioning 10 μM sections, slices were imaged on Zeiss AX10 fluorescence microscope to detect Dil signals in brain cells.

Group setting and intravenous administration of EVs to 5×FAD

Four-month-old 5×FAD mice were randomly divided into 3 groups: 5×FAD + PBS (n = 10/group), 5×FAD + NSC-EVs (n = 11/group), and 5×FAD + iNSC-EVs (n = 11/group). Same age C57BL/6 mice injected with PBS (WT + PBS) were used as controls (n = 10/group). 200 μl EVs (0.5 μg/μl concentration) or an equal volume of PBS control were administrated intravenously over 5 min *via* tail vein every three days. Each animal received 10 injections. The dosage of EVs was chosen based on our recent stroke studies.^1^

Nanoparticle tracking analysis (NTA)

NTA analyses were carried out to determine the sizes and concentrations of EV samples. Briefly, EVs were resuspended in 1 ml PBS for NTA analyses at defined conditions of the measurements (25 °C, 1 cP viscosity, 25 sec per capture frame, and 60 sec measurement time). NTA analyses were assessed on NanoSight NS300 system (Malvern Instruments, UK) with a sCMOS camera. Three individual measurements were applied for measuring the sizes and concentration of EVs.

Electron microscopy (EM)

For scanning electron microscopy (SEM), NSCs and iNSCs cultured on glass coverslips were fixed with 2.5% glutaraldehyde and washed three times with PBS. Cells were dehydrated in a series of increasing ethanol concentrations and transferred for critical drying. Cells were then coated with gold-palladium to increase the image contrast and imaged using scanning electron microscope (S-3400, Hitachi). For transmission electron microscopy (TEM), purified EVs were negatively stained and then spread on the copper grids. The droplets of EVs were removed with filter paper and air-dried at RT. Images were taken using transmission electron microscopy (JEM-1230, JEOL Ltd.).

Morris Water Maze (MWM) test

MWM was performed to determine the cognitive function and memory of mice. Mice were introduced into a circular, water-filled tank which was equally divided into four quadrants. Visual cues were placed around the pool in plain sight of the mouse to flag the submerged platform. Various parameters of mouse movement were recorded, including the time spent in each quadrant of the pool, the time taken to reach the platform (escape latency), and the total distance travelled. For each trial, the mouse was allowed no more than 60 sec to find the submerged platform before they were guided to the platform, removed from water, towel dried, and returned to their cage. Each mouse completed 4 trials per day during the 6-day training phase. One day after the training, the probe test was conducted. The platform was removed and each mouse was given 60 sec to swim in the water. The swimming was videotaped and analyzed by Ethovision XT (Noldus, Netherlands).

Y maze

The Y-maze had three arms (20 cm long × 10 cm wide × 20 cm high) at 120° angles. The three arms included the start arm (always open); the novel arm, which is blocked at the first trial, but opened at the second trial; and the other arm (always open). In the first trial, one of the arms is blocked and the animal is allowed to explore the rest two arms for 10 min. The second trial was conducted 2 h after the first trial. In the second trial, the blocked arm is opened and considered as the novel arm. The animal is allowed to explore all three arms for 5 min and the entrance to each arm is recorded. The spatial memory of animals can be revealed by a discrimination to the novel arm in the second trial. A video camera linked to the Any-Maze animal tracking system software was mounted above the maze, and used to record the movements of the mice for analysis. The time spent in and entries into the novel arms indicated the spatial recognition memory (learned behavior). The experimental environment was kept quiet, light, slightly dark and each of the arms of the Y maze was cleaned with 70% ethanol solution between trials.

Open Field Test

Open field test was performed using an open field apparatus (30 × 30 × 21 cm) with nine virtual quadrants (10 × 10 cm each). Central area is a region consisting of four sub squares marked with red color. Each mouse was put in the center at start of test and permitted to freely explore for 5 min. Number of crossings, time spent in the center and periphery, rearing, fecal pallets, time of immobility, jumping, and efforts made by each mouse to getaway were recorded. The total distance and time as well as the distance travelled and time spent in the center zone (10 cm × 10 cm) were analyzed using the Activity Monitor software (Med Associates, Inc.). The apparatus was cleaned with 70% ethanol between trials.

Fear conditioning test

The test consists of a training phase and test phase, which took place on two consecutive days. During the training phase, mice were placed in the conditioning chamber for a 120 sec accommodation period. The mice were then presented with an 80 dB tone for 30 s, co-terminating with a two second 0.7 mA foot shock, followed by another 120 s interval and a second tone and foot-shock pairing. Mice were removed from the chamber thirty seconds after the second shock. In the context test, mice were placed in the same chamber used during training for 5 min without a tone or footshock, and the duration of freezing behavior was recorded using a stopwatch. The percentage freezing time (defined as the time in which mice had no movements except for respiration) was recorded and analyzed by the Panlab Startle and Fear combined system with Packwin 2.0 software.

Protein extraction and western blotting

Brains tissues were removed from euthanized mice and homogenized by a homogenizer in the M-PER Protein Extraction Buffer (Pierce) containing a protease inhibitor cocktail (Sigma). Protein concentrations were determined using BCA Protein Assay Kit (Pierce). Proteins (5~10 μg) from tissue lysates were separated by sodium dodecyl sulfate-polyacrylamide gel electrophoresis (SDS-PAGE) and electrophoretic transferred to polyvinyldifluoridene membranes (Millipore and Bio-Rad). Proteins were treated with purified primary antibodies for CD9 (rabbit, Abcam, 1:2000), Flotillin1 (mouse, BD Biosciences; 1:5000), Flotillin2 (rabbit, CST, 1:1000), APOA1 (rabbit, Affinity Biosciences; 1:500), APOA2 (rabbit, Affinity Biosciences; 1:500), CD68 (rabbit, Abcam; 1:1000), CD86 (rabbit, CST; 1:1000), or β-actin (mouse, CST; 1:1000) overnight at 4°C followed by a horseradish peroxidase-linked secondary anti-rabbit or anti-mouse antibody (Cell Signaling Technologies, 1:10,000). Antigen-antibody complexes were visualized by Pierce ECL Western Blotting Substrate (Thermo Fisher Scientific, Waltham, MA). Films were then scanned with a CanonScan 9950F scanner and the acquired images were analyzed on a Macintosh computer using the free public domain NIH ImageJ program (at <http://rsb.info.nih.gov/nih-image/>).

Enzyme-linked immunosorbent assay (ELISA)

Mouse hippocampal and cortical tissue lysates were collected and commercially available ELISA kits were used to measure the levels of Aβ_1-42_ (cat# KE1266, Immunoway), TNF-α (cat# KE1419, Immunoway), and IL-1β (cat# KE1416, Immunoway) according to manufacturer’s protocols. Diluted standards and lysates in triplicate were added to the corresponding wells and incubated at RT for 2 h on a microplate shaker. Sample Diluent was used as blank control. Streptavidin-HRP was added (100 μl/well) and incubated at RT for 45 min. TMB Substrate Solution was then added (100 μl/well) and incubated at RT for 30 min. The enzyme reaction was stopped by Stop Solution (100 μl/well). Absorbance of each well was read using spectrophotometer DV8200 (Drawell). Aβ_1-42_, TNF-α, and IL-1β concentrations were determined according to the standard curve.

Immunohistochemistry

Tissue sections were fixed in 4% paraformaldehyde (Sigma) overnight at 4°C and transferred into 30% sucrose to incubate at 4 °C for 24 h. Post 3 times of PBS washes, sections were incubated with permeabilizing and blocking buffer containing 5% goat serum (Vector Laboratories) and 0.5% Triton X-100 (Bio-Rad) in PBS at RT for 1 h. Tissue sections were then incubated with primary antibody for Aβ (mouse, cat# 15126S, CST, 1:400), Nestin (chicken, cat# NB100-1604, Novus, 1:5000), Sox2 (Rabbit, cat#ab92494, Abcam, 1:500), CD9 (rabbit, cat#98327, CST, 1:100), Ki67 (rabbit, cat# 9129S, CST, 1:400), DCX (rabbit, cat# 4604S, CST, 1:400), NeuN (mouse, cat# MAB377, Millipore, 1:200), or Iba1 (goat, cat# ab5076, Abcam, 1:500) overnight at 4 °C. The next day, tissue sections were incubated with secondary antibodies (Molecular Probes) at RT for 1 h after washing with PBS for 3 times. Sections were mounted using Vecta-Shield (Vector Laboratories). Images were taken using a Zeiss AX10 fluorescence microscope accompanied with ZEN 2.3 (blue edition) software. For quantification of immunoreactivity positive cell density, the hippocampus, prefrontal cortex (PFC), and sub-ventricular zone (SVZ) were imaged at ×20 magnification. Three coronal sections spanning the hippocampus, PFC, and SVZ at different depths on the rostro-caudal axis were analyzed for each animal. Four to five images were captured on matching areas of selected brain regions per section. The numbers of immunoreactivity positive amyloid plaques and cells were quantified by ImageJ. The amyloid plaque burden (area occupied by all plaques divided by the total area) was estimated in the cortex and hippocampus for each section using the Analyze Particles plugin of ImageJ software. Values from each section were averaged to acquire a mean immunoreactivity positive amyloid plaque and cell density for each animal.

Golgi-Cox staining

Mouse cortical and hippocampal tissues were incubated at RT in Golgi-Cox staining solution A/B of FD Rapid Golgi Stain™ Kit (FD Neurotechnologies) for 14 d protected from light. The solution C was replaced the following day and brain tissues were incubated in solution C at 4 °C for 72 h in the dark. Afterwards, brain tissue blocks were included in a 10% sucrose-4% agarose solution, obtaining 100 µm thick sections in a Leica VT1200S vibratome. Sections were mounted on gelatin-coated glass slides and naturally dried for 20 min. The Golgi-Cox reaction was performed following the manufacturer’s instructions. Sections were finally counterstained with toluidine blue to identify anatomical structures. Images were taken at 20, 40, and 64 objectives using Olympus1X71.

Quantitative Reverse Transcription Polymerase Chain Reaction (qRT-PCR)

The mRNAs and miRNAs were isolated from brain tissues and cells using RNeasy mini kit (Qiagen) according to the manufacturer’s instructions. Genomic DNA was removed using DNase I digestion kit (Qiagen). cDNA was synthesized using miScript II RT kit (Qiagen). Transcripts were amplified using SYBR green PCR kit (Qiagen) with the ABI7500 (Applied Biosystems) with specific primer sets for *Gapdh* (Forward: CATGTTCCAGTATGACTCCACTC, Reverse: GGCCTCACCCCATTTGATGT), *Il1b* (Forward: CCAGCAGGTTATCATCATCATCC, Reverse: CTCGCAGCAGCACATCAAC), *Nos2* (Forward: CCCTTCAATGGTTGGTACATGG, Reverse: ACATTGATCTCCGTGACAGCC), *Tnf* (Forward: ACGTGGAACTGGCAGAAGAG, Reverse: GGTCTGGGCCATAGAACTGA), *Myod88* (Forward: CGGGTCCCTGGACTCCTTCA, Reverse: CGCGTTTCCAGCTCTCGGAT), *Ym1* (Forward: TCACAGGTCTGGCAATTCTTCTG, Reverse: ACTCCCTTCTATTGGCCTGTCC), *CD206* (Forward: TCTTTGCCTTTCCCAGTCTCC, Reverse: TGACACCCAGCGGAATTTC), and miRNAs (Universal primer: GAATCGAGCACCAGTTACGC, U6 primer: TGGCCCCTGCGCAAGGATG, miR-9: TCTTTGGTTATCTAGCTGTATGA, let-7i: TGAGGTAGTAGTTTGTGCTGTT, miR-10b: TACCCTGTAGAACCGAATTTGTG, miR-21a: TAGCTTATCAGACTGATGTTGA, miR-30a: TGTAAACATCCTCGACTGGAAG, miR-99a: AACCCGTAGATCCGATCTTGTG, let-7f: TGAGGTAGTAGATTGTATAGTT, let-7g: TGAGGTAGTAGTTTGTACAGTT, miR-181a: AACATTCAACGCTGTCGGTGAGT, miR-26a: TTCAAGTAATCCAGGATAGGCT, miR-129: CTTTTTGCGGTCTGGGCTTGC, let-7c: TGAGGTAGTAGGTTGTATGGTT, let-7b: TGAGGTAGTAGGTTGTGTGGTT, miR-148a: TCAGTGCACTACAGAACTTTGT, miR-100: AACCCGTAGATCCGAACTTGTG). Reactions were run in triplicates for each sample and no-template blanks were used as negative controls. Values were normalized to the *Gapdh* (for mRNA) and *U6* snRNA (for miRNA).

RNA sequencing (RNA-seq)

Total RNA was extracted from cortical and hippocampal tissues of experiment and control mice using the mirVana miRNA Isolation Kit (Ambion). Sample processing was carried out by OE Biotech Co., Ltd (Shanghai, China). RNA integrity was evaluated using the Agilent 2100 Bioanalyzer (Agilent Technologies). The libraries were constructed using TruSeq Stranded mRNA LTSample Prep Kit (Illumina). Libraries were sequenced on the Illumina sequencing platform (Illumina NovaSeq 6000) and 125bp/150bp paired-end reads were generated. Raw data (raw reads) were processed using Trimmomatic. Clean reads were mapped to reference genome using hisat 2. FPKM value of each gene was calculated using cufflinks and the read counts of each gene were obtained by htseq-count DEGs were identified using the DESeq 2012 R package functions estimateSizeFactors and nbinomTest. P value < 0.05 and foldChange > 2 or foldChange < 0.5 was set as the threshold for significantly differential expression Hierarchical cluster analysis of differentially expressed genes (DEGs) was performed to explore genes expression pattern. The enrichment of Gene Ontology (GO) term and Kyoto Encyclopedia of Genes and Genomes (KEGG) pathways in DEGs were analyzed using DAVID online tools (david.abcc.ncifcrf.gov). GO terms corresponding to biological process were selected. The enrichment of GO terms and KEGG pathways was determined by the *p*-value from DAVID online tool analysis. The *p*-value were determined by a modified Fisher's exact test, adjusted by the Benjamini-Hochberg method.

miRNA microarray

Total RNA was extracted from NSC-EVs and iNSC-EVs. Sequencing libraries were generated from 3 μg total RNA per sample as input material using NEBNext® Multiplex Small RNA Library Prep Set for Illumina® (NEB). The clustering of the index-coded samples was performed on a cBot Cluster Generation System using TruSeq SR Cluster Kit v3-cBot-HS (Illumia). After cluster generation, the library was sequenced on an Illumina Hiseq 2500/2000 platform, and 50bp single-end reads were generated. Raw data (raw reads) of fastq format were firstly processed through custom perl and python scripts for quality control. The small RNA tags were mapped to reference sequence by Bowtie without mismatch to analyze their expression and distribution on the reference. Mapped small RNA tags were used to match with known miRNA. miRBase 20.0 was used as reference for known miRNA, miRDeep2 and sRNA-tools-cli were used to obtain novel miRNAs and draw the secondary structures, respectively. miRNA expression levels were estimated by TPM (transcript per million).

Statistical analyses

The statistical difference between two independent groups was analyzed with the unpaired Student’s *t*-test, and that among more than two groups was assessed with the parametric one-way ANOVA with post-hoc Bonferroni test. Data were shown as mean ± s.d., and significance was determined as *p* < 0.05.

**
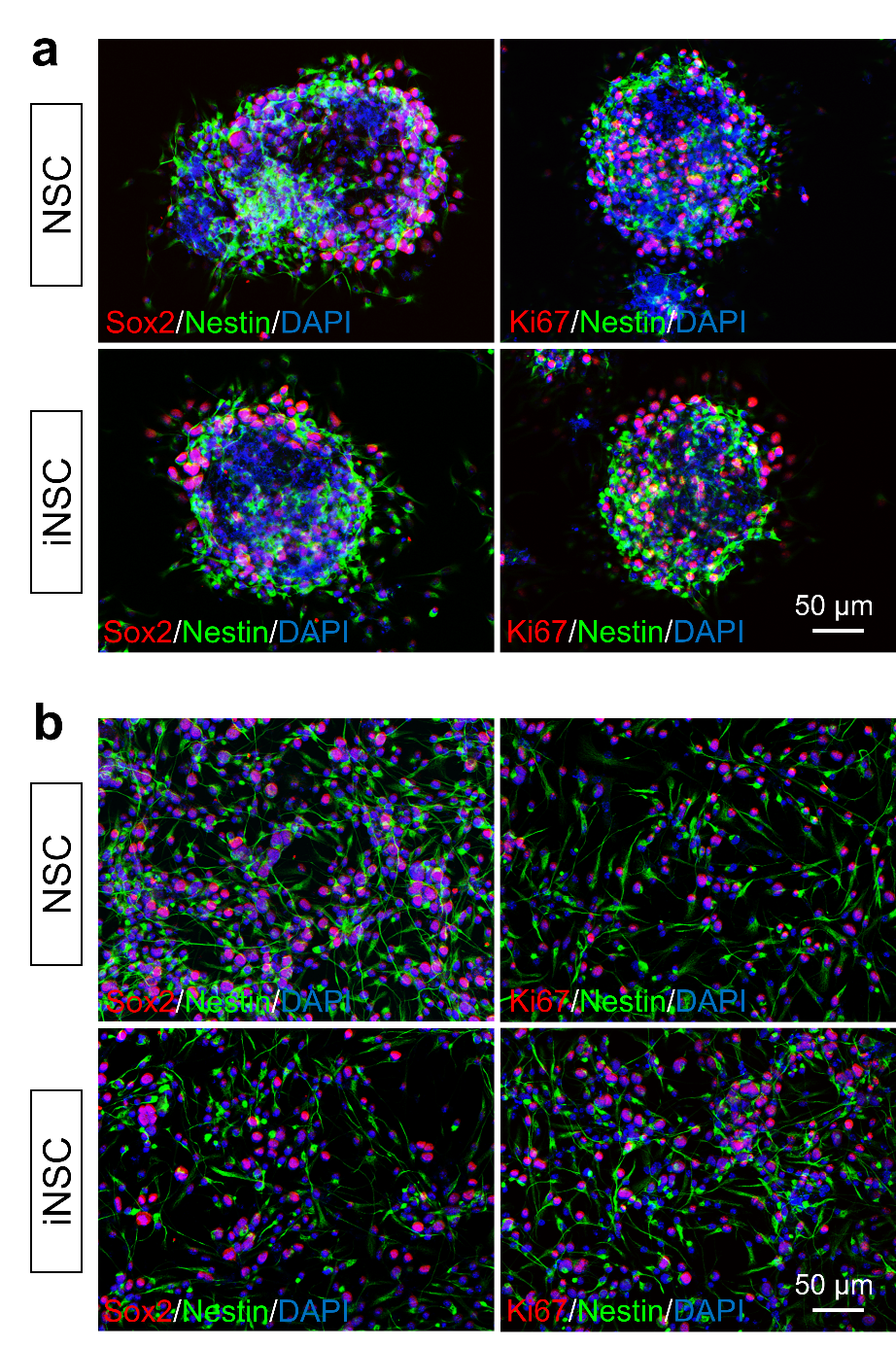
**

**Fig. S1. Characterization of NSCs and iNSCs.**

(**a**) Representative confocal microscopy images of Sox2, Nestin, and Ki67 immunoreactivities in the neurospheres generated by NSCs and iNSCs. (**b**) Representative confocal microscopy images of Sox2, Nestin, and Ki67 immunoreactivities in NSCs and iNSCs in adherent cell culture. Scale bar: 50 μm.

**
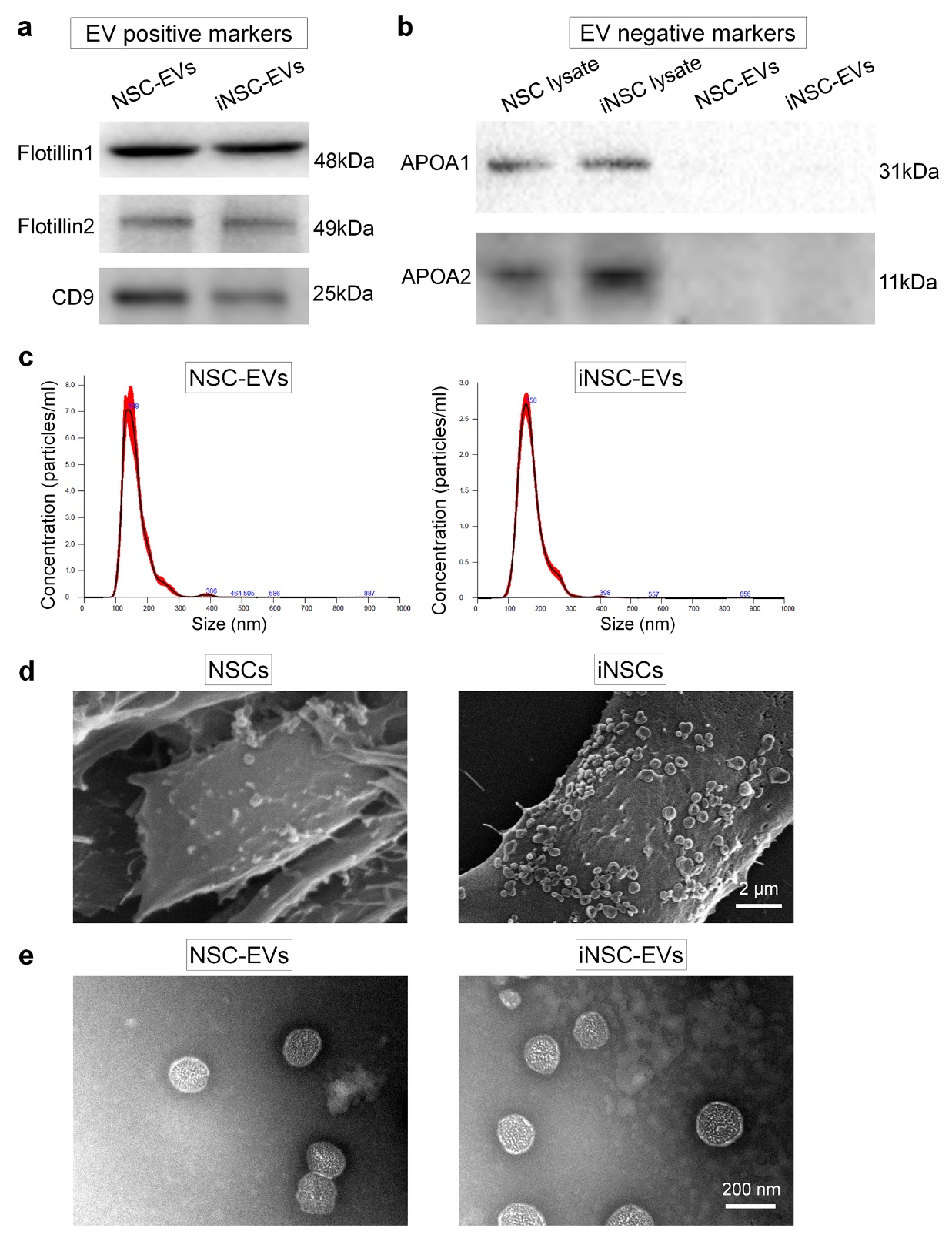
**

**Fig. S2. Characterization of NSC-EVs and iNSC-EVs.**

(**a**) Representative blots of positive EV markers Flotillin1, Flotillin2, and CD9 in NSC- and iNSC-EVs. (**b**) Representative blots of negative EV markers APOA1 and APOA2 in EVs and their parent cells. (**c**) NTA analysis of NSC- and iNSC-EVs. (**d**) SEM of NSCs and iNSCs. (**e**) TEM characterization of the morphology of NSC- and iNSC-EVs. Scale bar: 2 μm (**d**), and 200nm (**e**).

**
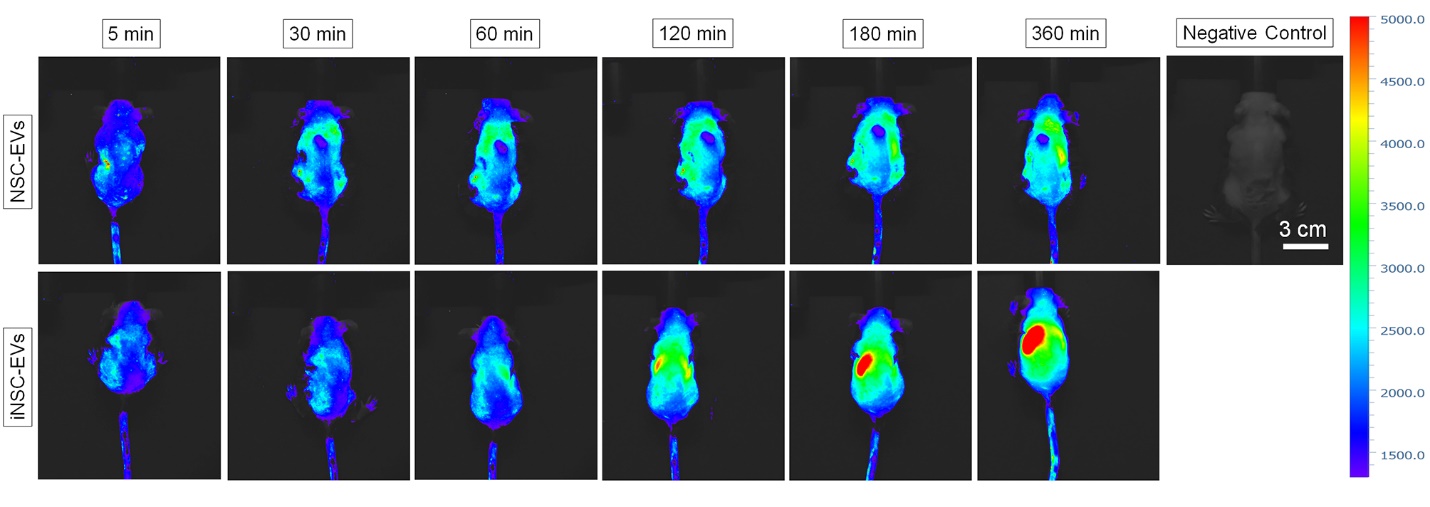
**

**Fig. S3. *In vivo* imaging of Dil-labeled NSC-EVs and iNSC-EVs after intravenous administration**.

Mice were injected intravenously with Dil-labeled EVs. The distribution of Dil-labeled EVs was observed by *in vivo* fluorescence imaging, showing distributed Dil-labeled EVs throughout the whole bodies of mice from 5 min to 360 min after EVs injection. PBS-injected mice were used as negative controls.


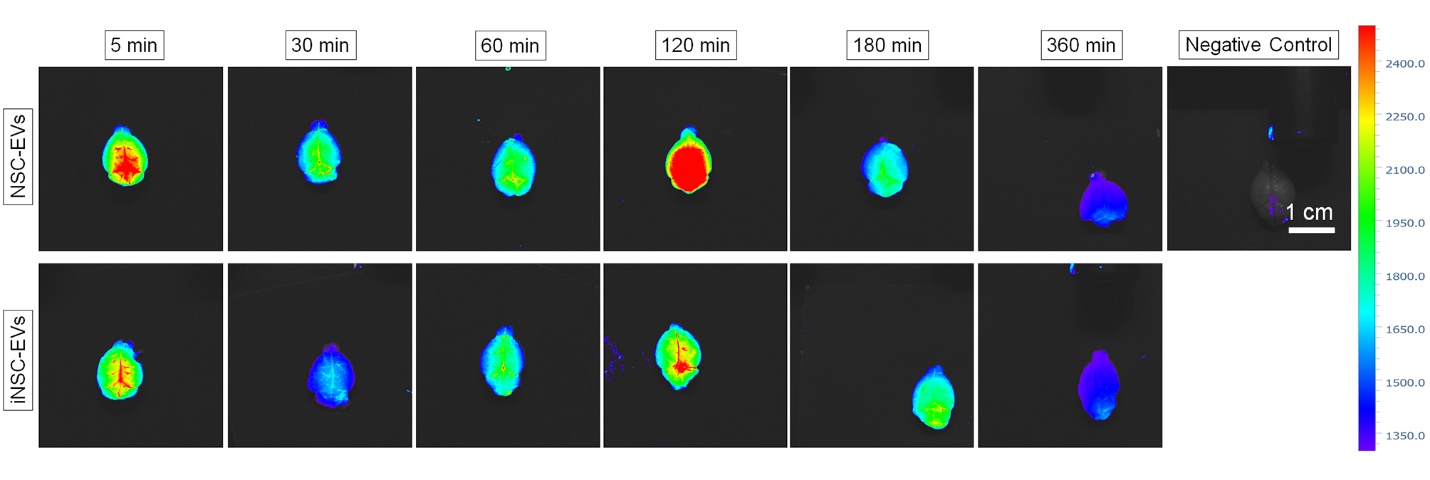


**Fig. S4. Fluorescence images of mouse brain tissues after intravenous administration of Dil-labeled NSC-EVs and iNSC-EVs**.

Mouse brains were dissected out at different time points after intravenous injection of Dil-labeled EVs. Brains of PBS-injected mice were used as negative controls.


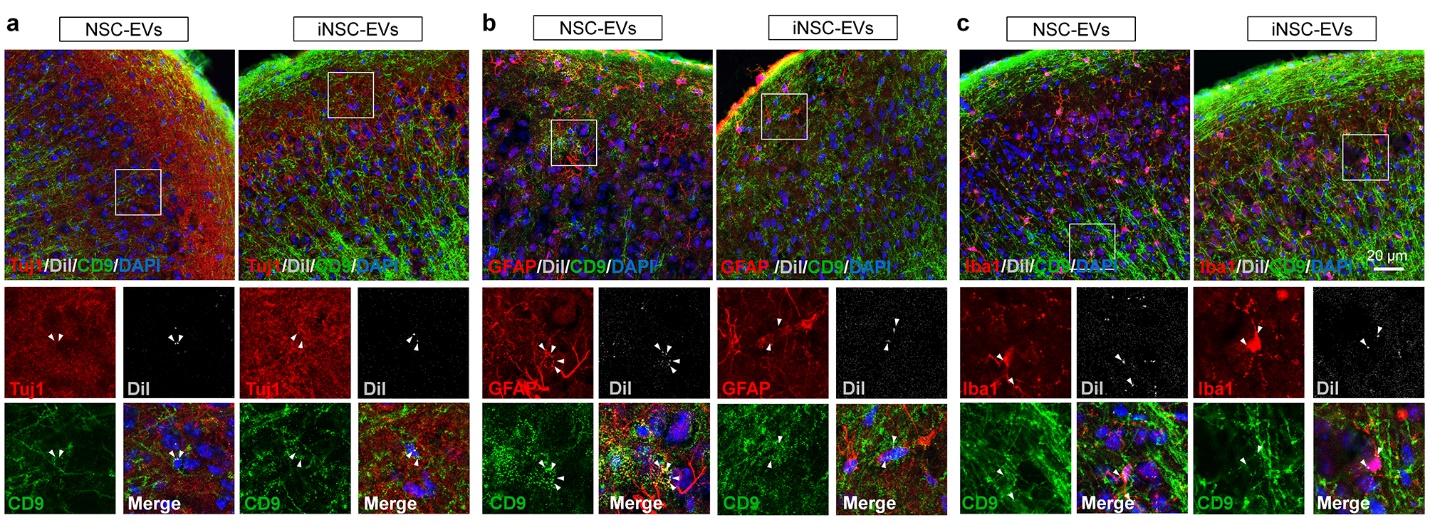


**Fig. S5. The uptake of NSC-EVs and iNSC-EVs by cortical cells after intravenous administration**.

Mice were injected intravenously with Dil-labeled EVs. Brain tissues were collected 5 min after EVs injection. (**a**) Representative confocal microscopy images of Tuj1, CD9, and Dil immunoreactivities in the cortex. (**b**) Representative confocal microscopy images of GFAP, CD9, and Dil immunoreactivities in the cortex. (**c**) Representative confocal microscopy images of Iba1, CD9, and Dil immunoreactivities in the cortex. Images at the bottom panels were high-magnification images of the corresponding small box area from the top panels in each group. Arrows indicate overlapping signals. Scale bar: 20 μm.


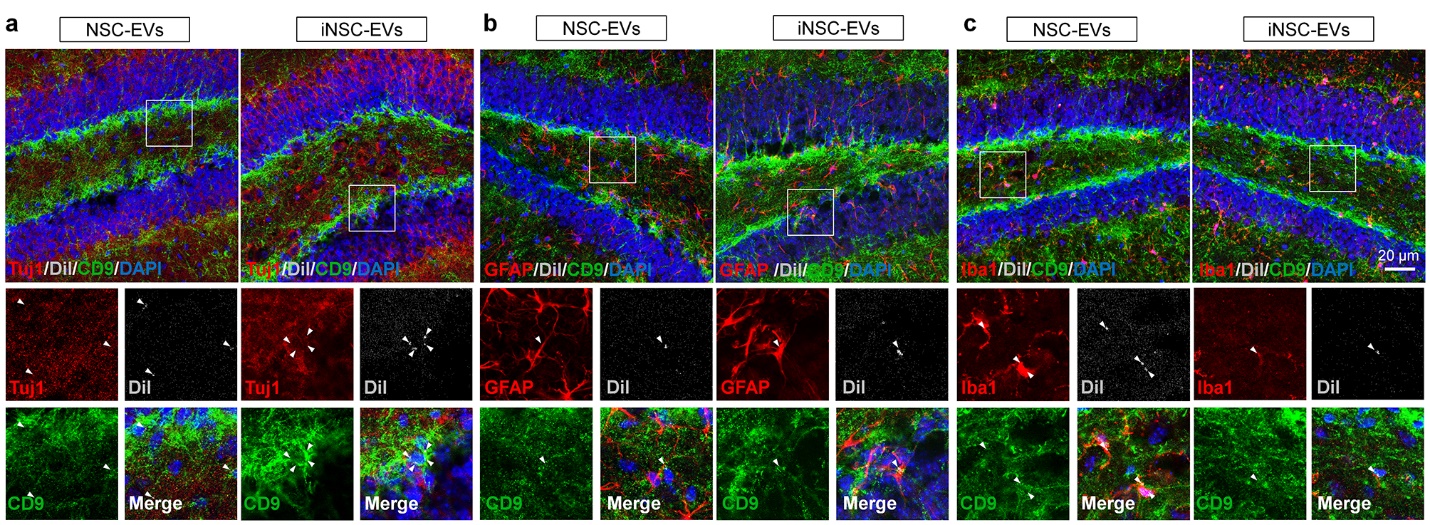


**Fig. S6. The uptake of NSC-EVs and iNSC-EVs by hippocampal cells after intravenous administration**.

Mice were injected intravenously with Dil-labeled EVs. Brain tissues were collected 5 min after EVs injection. (**a**) Representative confocal microscopy images of Tuj1, CD9, and Dil immunoreactivities in the hippocampus. (**b**) Representative confocal microscopy images of GFAP, CD9, and Dil immunoreactivities in the hippocampus. (**c**) Representative confocal microscopy images of Iba1, CD9, and Dil immunoreactivities in the hippocampus. Images at the bottom panels were high-magnification images of the corresponding small box area from the top panels in each group. Arrows indicate overlapping signals. Scale bar: 20 μm.


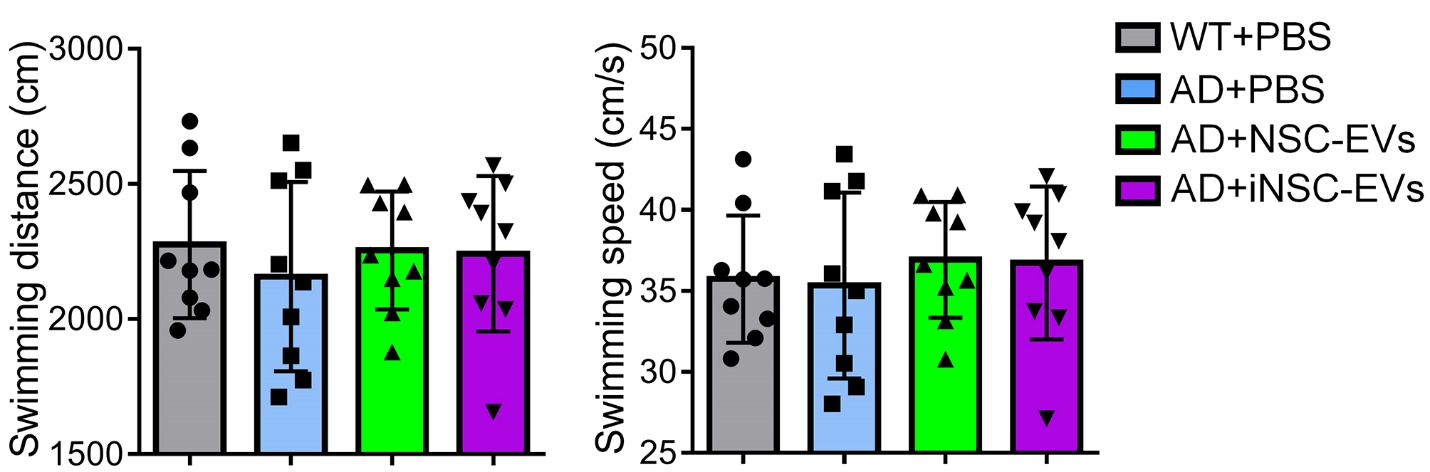


**Fig. S7.** **Intravenous administration of NSC-EVs and iNSC-EVs dose not alter motor functions of 5×FAD mice in Morris water maze.**

The total swimming distance and average swimming speed of mice in Morris water maze (n = 9). The statistical difference among groups was assessed with the parametric one-way ANOVA with post-hoc Bonferroni test.


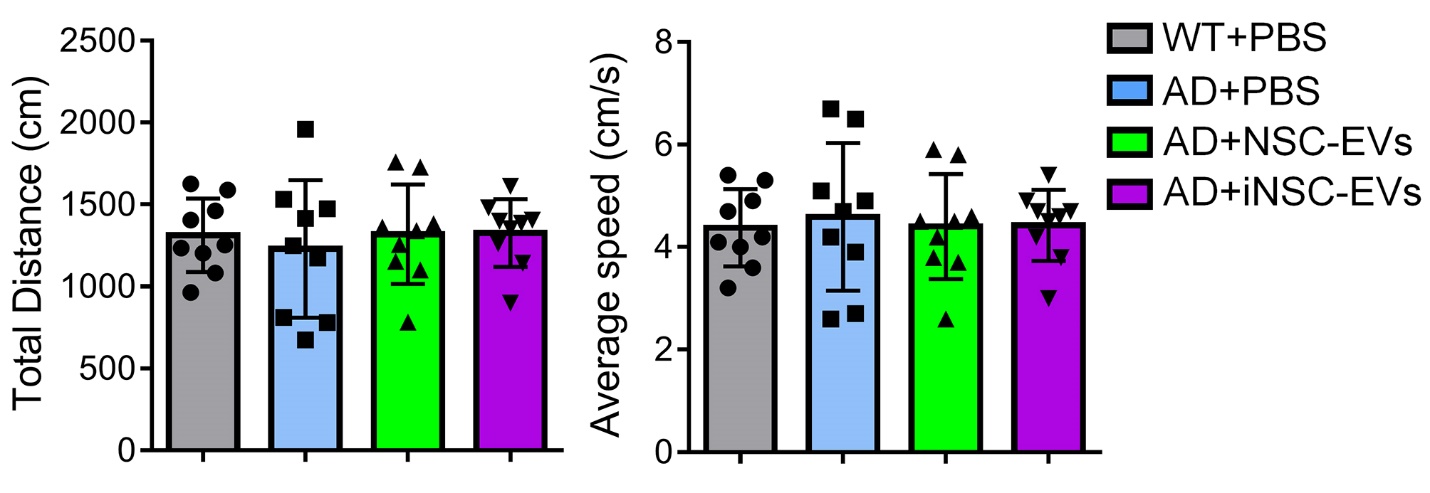


**Fig. S8.** **Intravenous administration of NSC-EVs and iNSC-EVs does not alter motor ability of 5×FAD mice in Y maze test.**

The total travel distance and average speed of mice in Y maze test (n = 9). The statistical difference among groups was assessed with the parametric one-way ANOVA with post-hoc Bonferroni test.


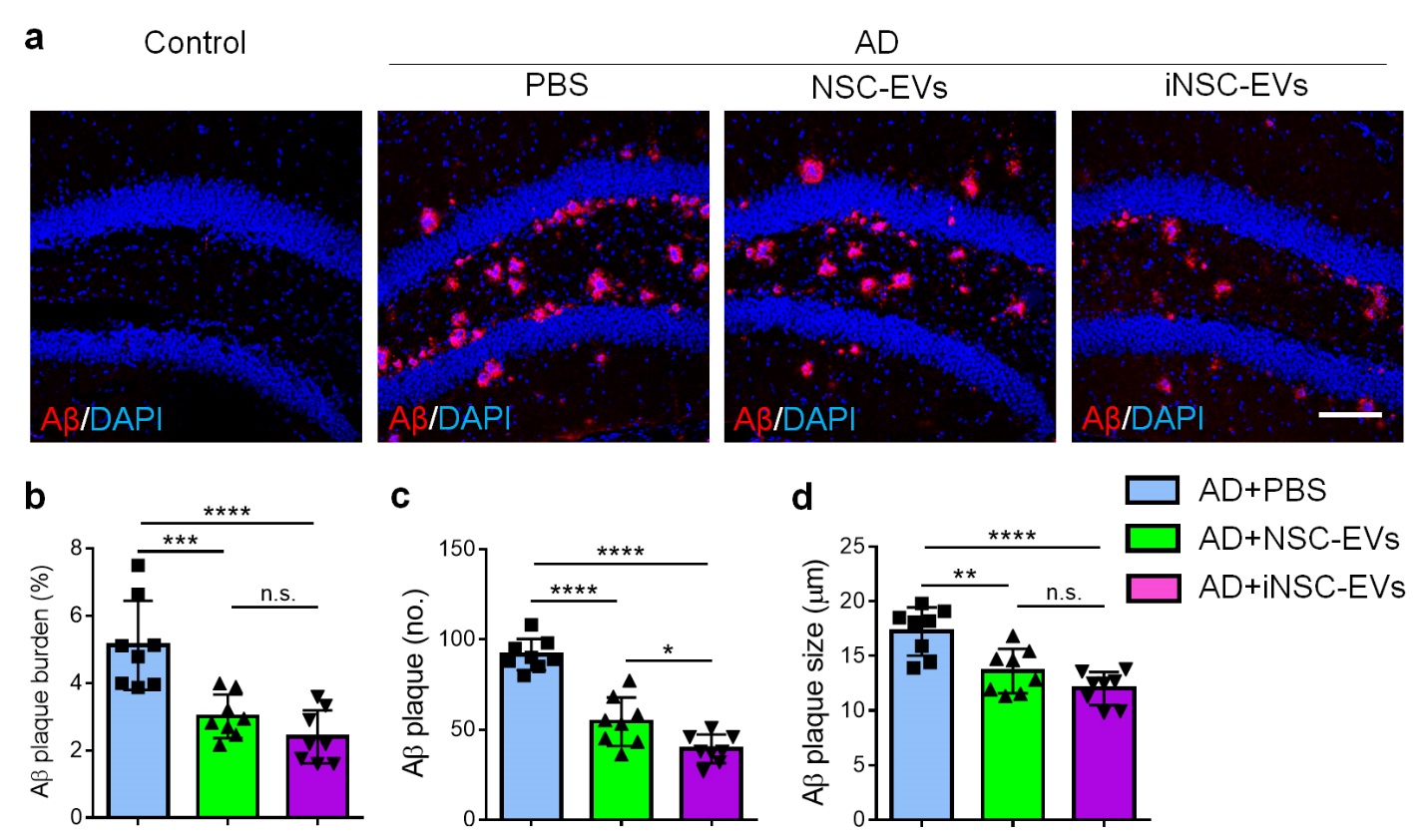


**Fig. S9.** **Intravenous administration of NSC-EVs and iNSC-EVs mitigates Aβ plaque deposition in the hippocampi of 5×FAD mice.**

(**a**) Representative confocal microscopy images of Aβ immunoreactivity in the hippocampus at ×20 magnification. (**b-d**) Aβ plaque burden (**b**), density (**c**), and average size (**d**) were quantified using ImageJ (n = 4). Scale bar: 200 μm. Error bars denote s.d.. *, **, ***, **** denote *p* < 0.05, *p* < 0.01, *p* < 0.001, and *p* < 0.0001, respectively. The statistical difference among groups was assessed with the parametric one-way ANOVA with post-hoc Bonferroni test.


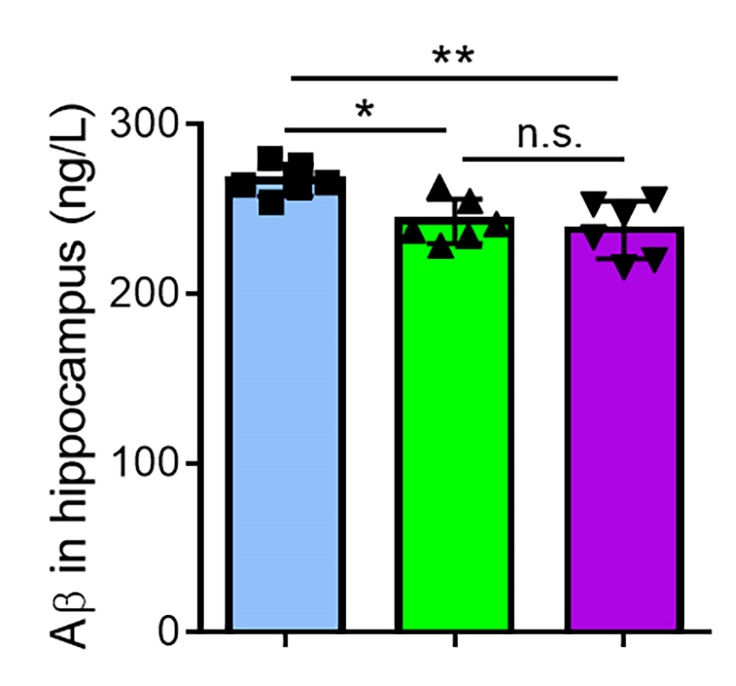


**Fig. S10.** **Intravenous administration of NSC-EVs and iNSC-EVs reduces Aβ accumulation in the hippocampi of 5×FAD mice.**

The levels of Aβ_1-42_ in the hippocampus were determined by ELISA assay (n = 6). Error bars denote s.d.. * and ** denote *p* < 0.05 and *p* < 0.01, respectively. The statistical difference among groups was assessed with the parametric one-way ANOVA with post-hoc Bonferroni test.


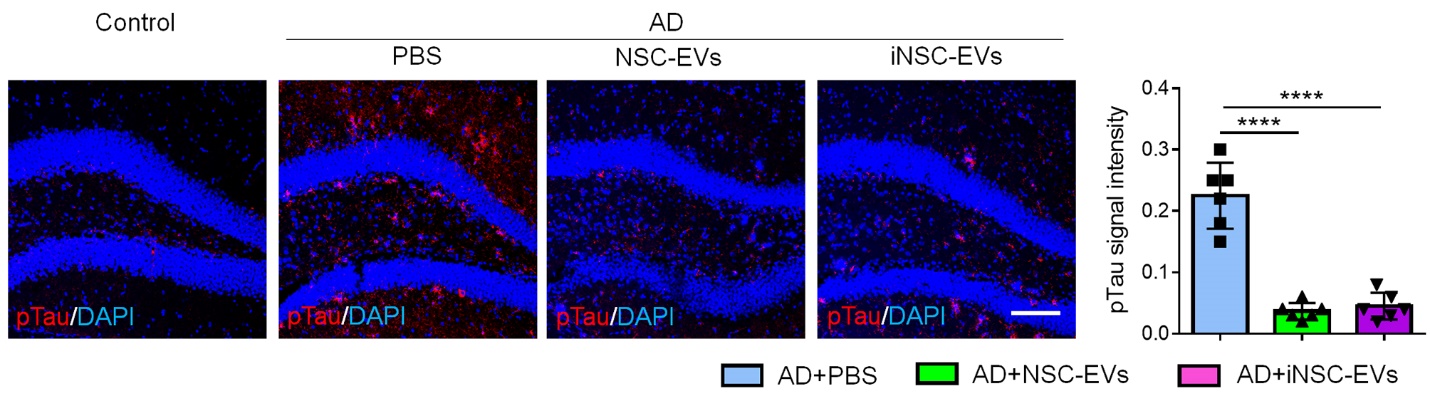


**Fig. S11.** **Intravenous administration of NSC-EVs and iNSC-EVs alleviates pTau propagation in the hippocampi of 5×FAD mice.**

Representative confocal microscopy images of pTau immunoreactivity in the hippocampus at ×20 magnification. The intensity of pTau signal was quantified using ImageJ (n = 6). Scale bar: 200 μm. Error bars denote s.d.. **** denotes *p* < 0.0001. The statistical difference among groups was assessed with the parametric one-way ANOVA with post-hoc Bonferroni test.


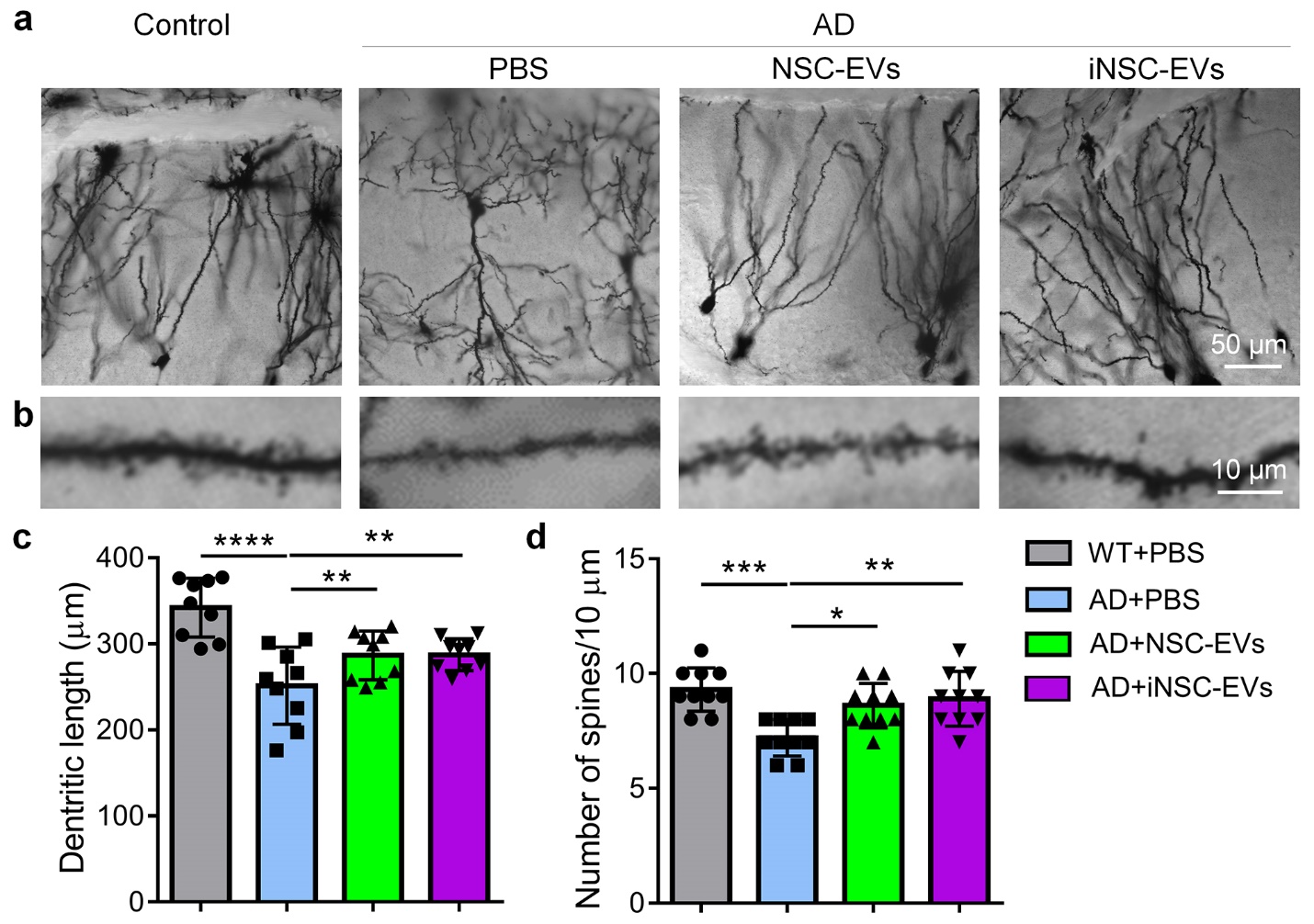


**Fig. S12.** **Intravenous administration of NSC-EVs and iNSC-EVs restores dendritic length and spine density in the hippocampi of 5×FAD mice.**

(**a**) Representative microscopy images of Golgi-Cox staining of hippocampal tissue samples at ×40 magnification. (**b**) Enlarged images demonstrating dendrites and spines. (**c**) Quantification of dendritic length of neurons in hippocampal tissue samples (n = 9). (**d**) Quantification of spine density in hippocampal tissue samples (n = 10). Error bars denote s.d.. *, **, ***, **** denotes *p* < 0.05, *p* < 0.01, *p* < 0.001, *p* < 0.0001, respectively. The statistical difference among groups was assessed with the parametric one-way ANOVA with post-hoc Bonferroni test.


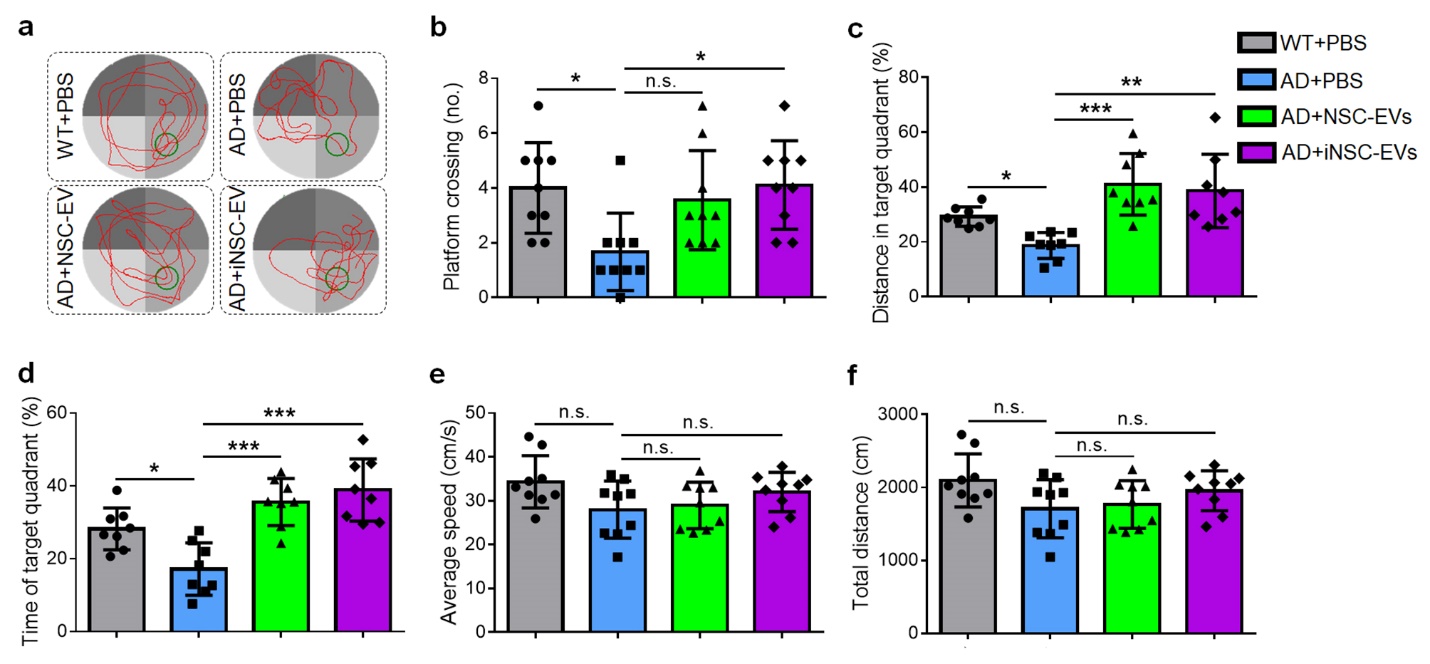


**Fig. S13.** **Intravenous administration of NSC-EVs and iNSC-EVs enhance cognitive performance of 5×FAD mice in Morris water maze.**

(**a**) Swim paths of mice while the platform was removed (memory phase); (**b**) Platform crossing numbers of mice in the target quadrant (n = 9). (**c**) Percentage of swimming distance (s) spent in the target quadrant of the Morris Water Maze (n = 9). (**d**) Percentage of swimming time (s) spent in the target quadrant of the Morris Water Maze (n = 9). (**e**) Average swimming speed (s) of mice in the Morris water maze test (n = 9). (**f**) Average swimming distance (s) of mice in the Morris water maze test (n = 9). Error bars denote s.d.. n.s. denotes non-significance. *, **, *** denote *p* < 0.05, *p* < 0.01, and *p* < 0.001, respectively. The statistical difference among groups was assessed with the parametric one-way ANOVA with post-hoc Bonferroni test.


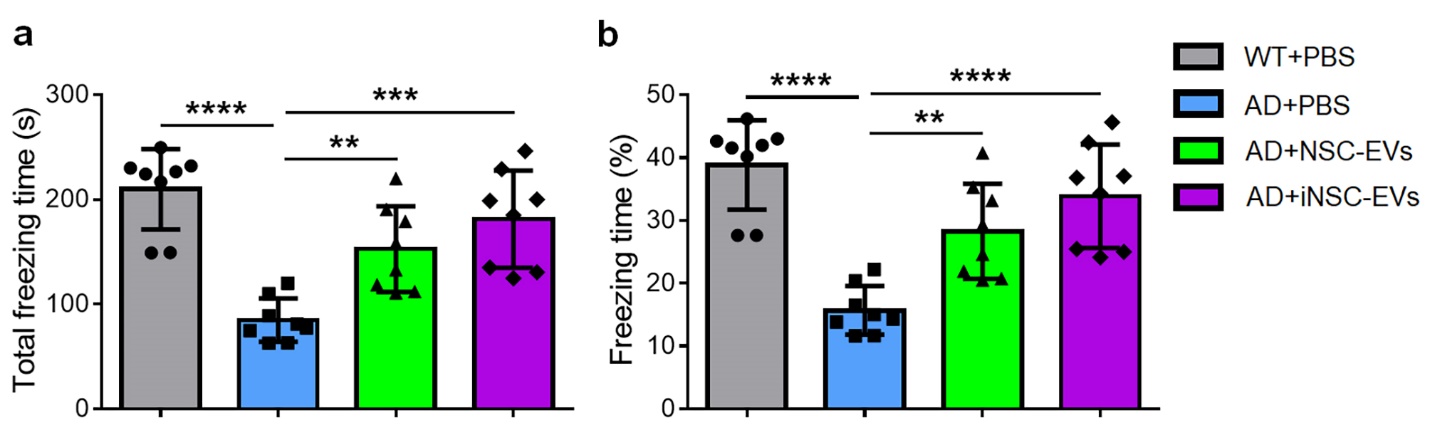


**Fig. S14.** **NSC-EVs and iNSC-EVs enhance cognitive performance of 5×FAD mice in fear conditioning test 1 month post intravenous administration.**

(**a**) Total freezing time (s) of mice in fear conditioning test. (**b**) Percentage of freezing time (s) of mice in fear conditioning test (n = 9). Error bars denote s.d.. n.s. denotes non-significance. **, ***, **** denote *p* < 0.01, *p* < 0.001, and *p* < 0.0001, respectively. The statistical difference among groups was assessed with the parametric one-way ANOVA with post-hoc Bonferroni test.


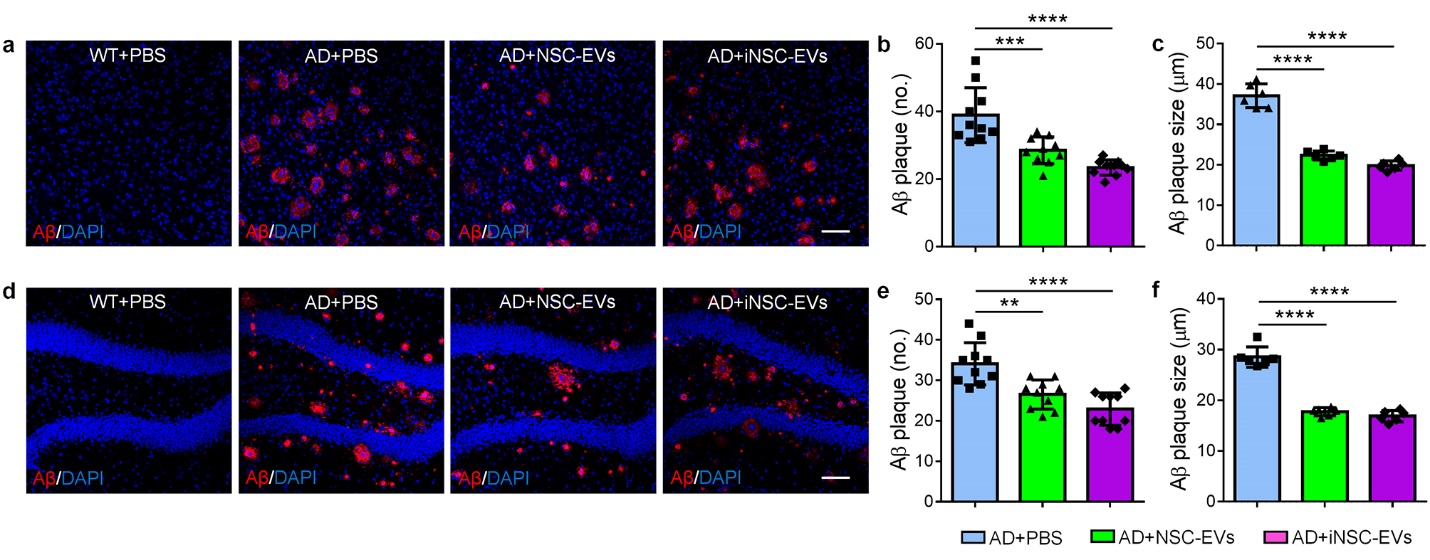


**Fig. S15.** **NSC-EVs and iNSC-EVs mitigated Aβ plaque deposition in the brains of 5×FAD mice 1 month post intravenous administration.**

(**a**) Aβ immunoreactivity in the PFC 1 month post EV administration. (**b, c**) Aβ plaque density (**b**) and average size (**c**) in the PFC were quantified using ImageJ (n = 9). (**d**) Aβ immunoreactivity in the hippocampal tissues 1 month post EV administration. (**e, f**) Aβ plaque density (**e**) and average size (**f**) in the hippocampal tissues were quantified using ImageJ (n = 9). Error bars denote s.d.. n.s. denotes non-significance. **, ***, **** denote *p* < 0.01, *p* < 0.001, and *p* < 0.0001, respectively. The statistical difference among groups was assessed with the parametric one-way ANOVA with post-hoc Bonferroni test.


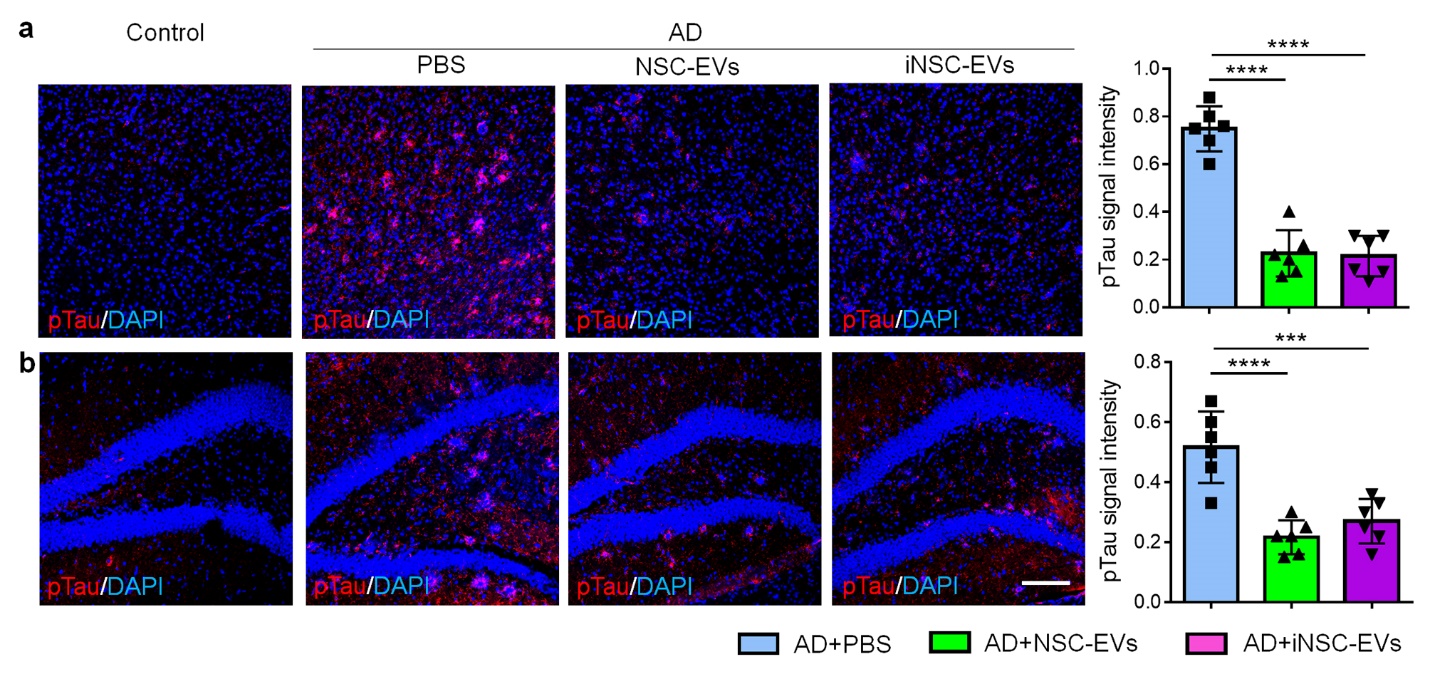


**Fig. S16.** **Intravenous administration of NSC-EVs and iNSC-EVs mitigates pTau propagation in the brains of 5×FAD mice.**

(**a**) Representative confocal microscopy images of pTau immunoreactivity in the PFC at ×20 magnification. The intensity of pTau signal quantified using ImageJ was provided on the right panel (n = 6). (**b**) Representative confocal microscopy images of pTau immunoreactivity in the hippocampus at ×20 magnification. The intensity of pTau signal quantified using ImageJ was provided on the right panel (n = 6). Scale bar: 200 μm. Error bars denote s.d.. *** and **** denote *p* < 0.001 and *p* < 0.0001. The statistical difference among groups was assessed with the parametric one-way ANOVA with post-hoc Bonferroni test.


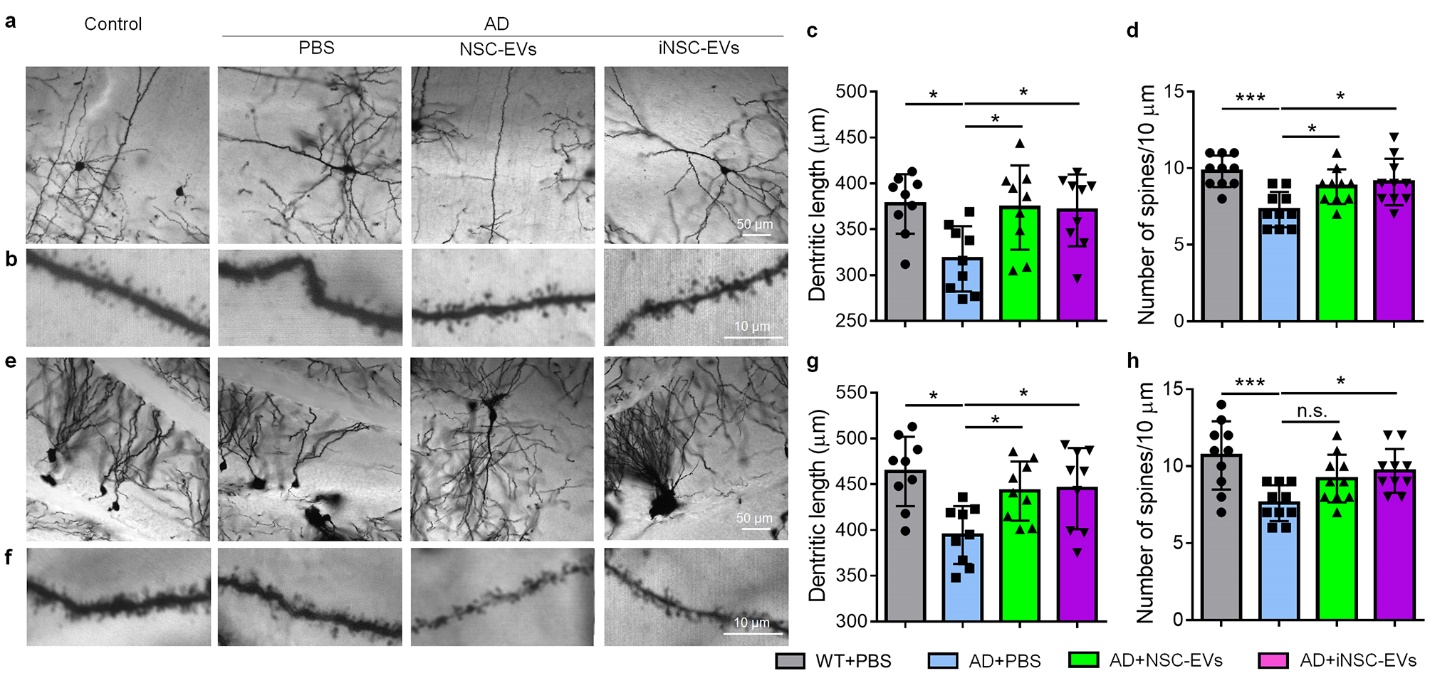


**Fig. S17.** **Intravenous administration of NSC-EVs and iNSC-EVs restores dendritic length and spine density in the brains of 5×FAD mice.**

(**a**) Representative microscopy images of Golgi-Cox staining of cortical tissue samples at ×40 magnification. (**b**) Enlarged images demonstrating dendrites and spines. (**c**) Quantification of dendritic length of neurons in cortical tissue samples (n = 9). (**d**) Quantification of spine density in cortical l tissue samples (n = 10). (**e**) Representative microscopy images of Golgi-Cox staining of hippocampal tissue samples at ×40 magnification. (**f**) Enlarged images demonstrating dendrites and spines. (**g**) Quantification of dendritic length of neurons in hippocampal tissue samples (n = 9). (**h**) Quantification of spine density in hippocampal tissue samples (n = 10). Error bars denote s.d.. * and *** denotes *p* < 0.05 and *p* < 0.001, respectively. The statistical difference among groups was assessed with the parametric one-way ANOVA with post-hoc Bonferroni test.


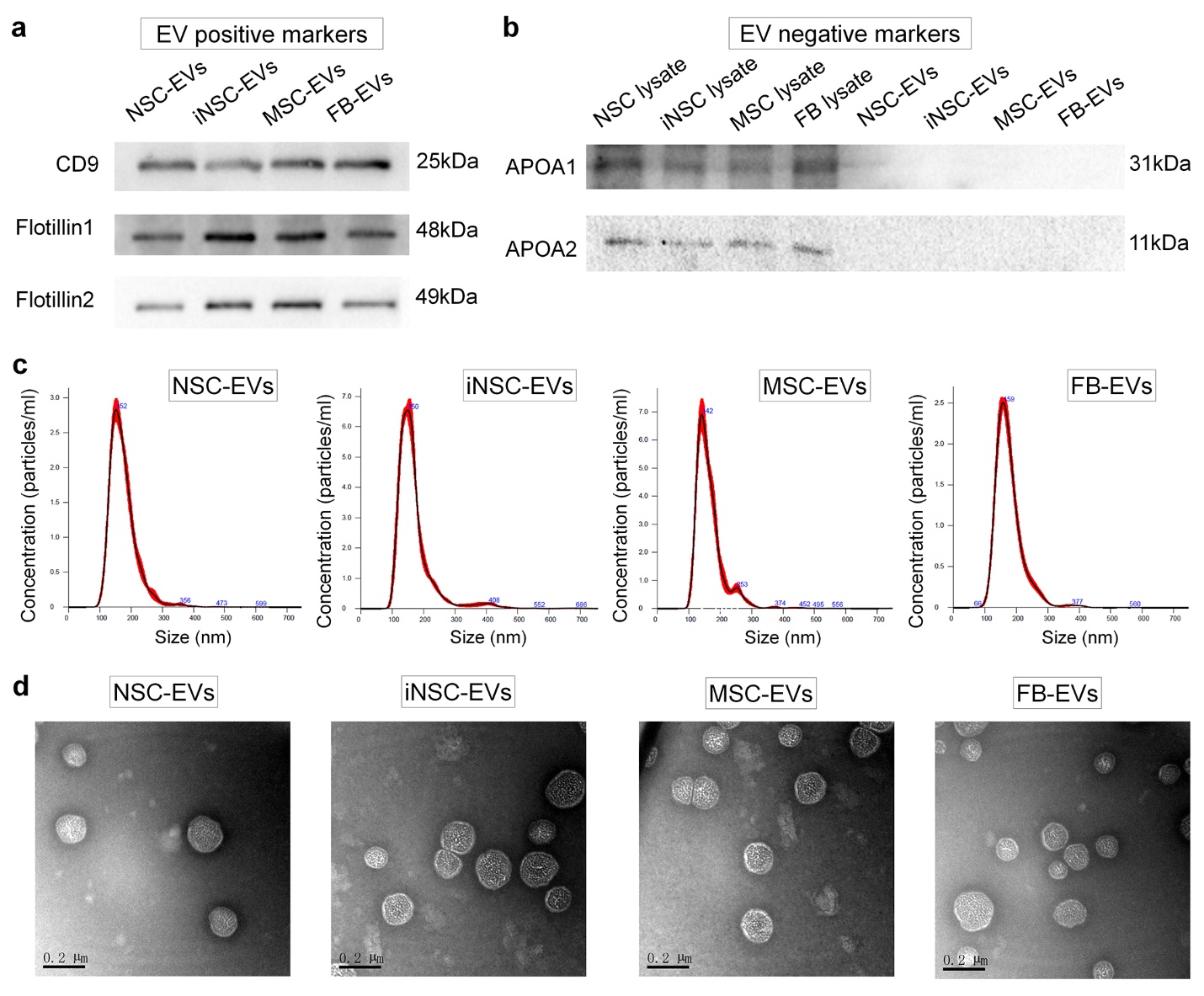


**Fig. S18. Characterization of NSC-EVs, iNSC-EVs, MSC-EVs, and FB-EVs.**

(**a**) Representative blots of positive EV markers Flotillin1, Flotillin2, and CD9 in NSC- and iNSC-EVs. (**b**) Representative blots of negative EV markers APOA1 and APOA2 in EVs and their parent cells. (**c**) NTA analysis of EVs. (**d**) TEM characterization of the morphology of EVs. Scale bar: 200nm (**e**).


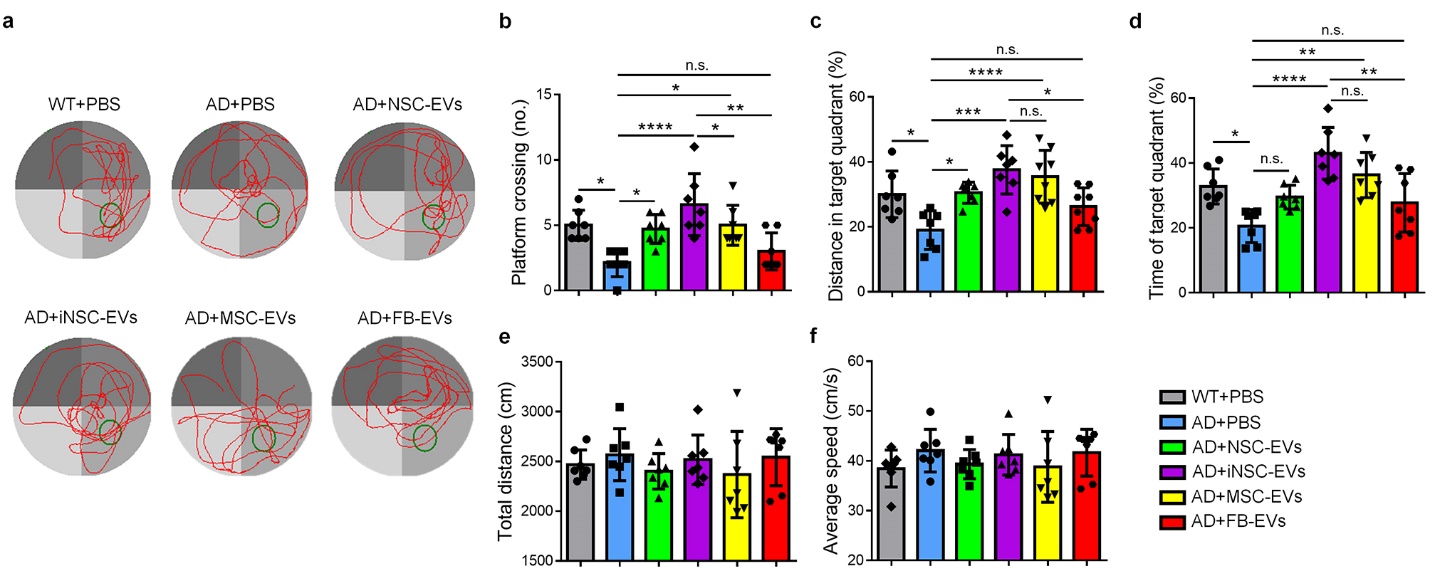


**Fig. S19.** **Intravenous administration of stem cell-derived EVs enhance cognitive performance of 5×FAD mice in Morris water maze.**

(**a**) Swim paths of mice while the platform was removed (memory phase); (**b**) Platform crossing numbers of mice in the target quadrant (n = 7). (**c**) Percentage of swimming distance (s) spent in the target quadrant of the Morris Water Maze (n = 7). (**d**) Percentage of swimming time (s) spent in the target quadrant of the Morris Water Maze (n = 7). (**e**) Average swimming speed (s) of mice in the Morris water maze test (n = 7). (**f**) Average swimming distance (s) of mice in the Morris water maze test (n = 7). Error bars denote s.d.. n.s. denotes non-significance. *, **, ***, **** denote *p* < 0.05, *p* < 0.01, *p* < 0.001, *p* < 0.0001, respectively. The statistical difference among groups was assessed with the parametric one-way ANOVA with post-hoc Bonferroni test.


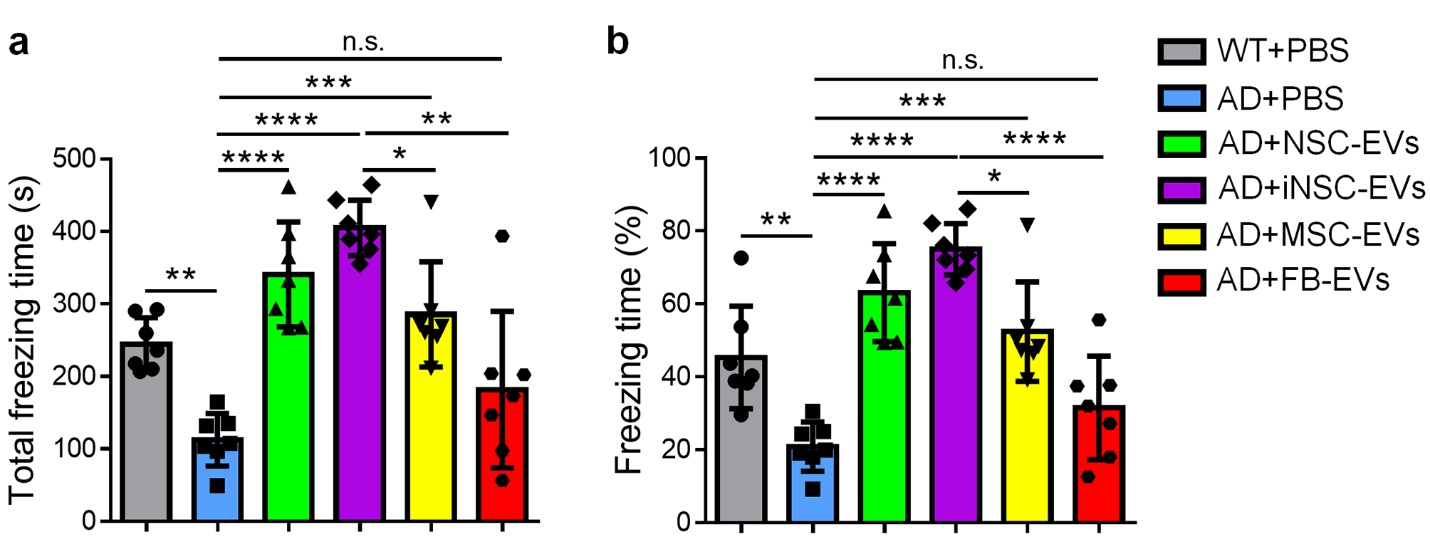


**Fig. S20.** **Intravenous administration of stem cell-derived EVs alleviated cognitive function of 5×FAD mice in fear conditioning test.**

(**a**) Total freezing time (s) of mice in fear conditioning test. (**b**) Percentage of freezing time (s) of mice in fear conditioning test (n = 9). Error bars denote s.d.. n.s. denotes non-significance. *, **, ***, **** denote *p* < 0.05, *p* < 0.01, *p* < 0.001, *p* < 0.0001, respectively. The statistical difference among groups was assessed with the parametric one-way ANOVA with post-hoc Bonferroni test.


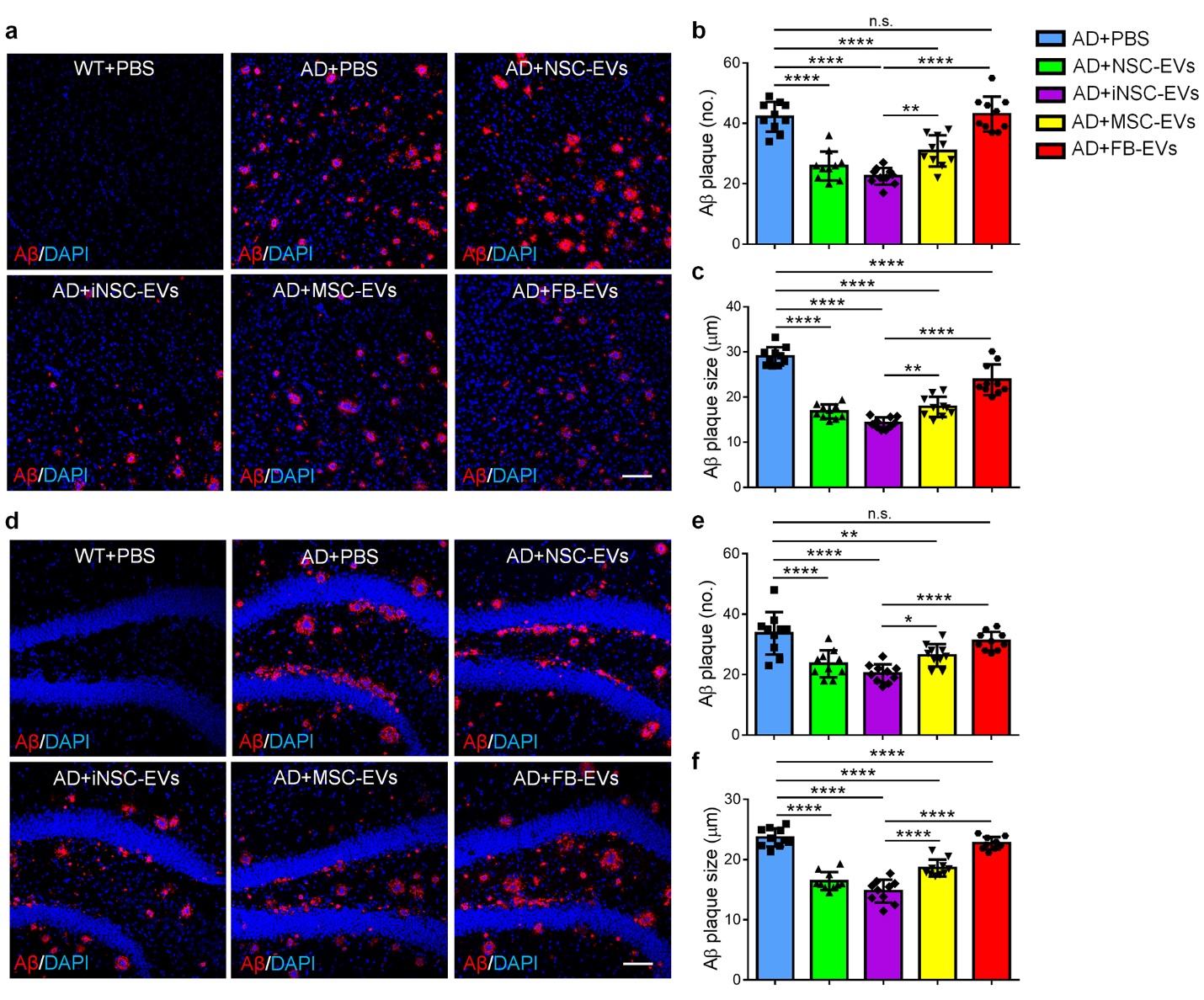


**Fig. S21.** **Intravenous administration of stem cell-derived EVs mitigates Aβ plaque deposition in the brains of 5×FAD mice.**

(**a**) Representative confocal microscopy images of Aβ immunoreactivity in the PFC at ×20 magnification. (**b, c**) Aβ plaque density (**b**) and average size (**c**) in the PFC were quantified using ImageJ (n = 10). (**d**) Representative confocal microscopy images of Aβ immunoreactivity in the PFC at ×20 magnification. (**e, f**) Aβ plaque density (**e**) and average size (**f**) in the hippocampus were quantified using ImageJ (n = 10). Scale bar: 200 μm. Error bars denote s.d.. *, **, **** denote *p* < 0.05, *p* < 0.01, *p* < 0.0001, respectively. The statistical difference among groups was assessed with the parametric one-way ANOVA with post-hoc Bonferroni test.


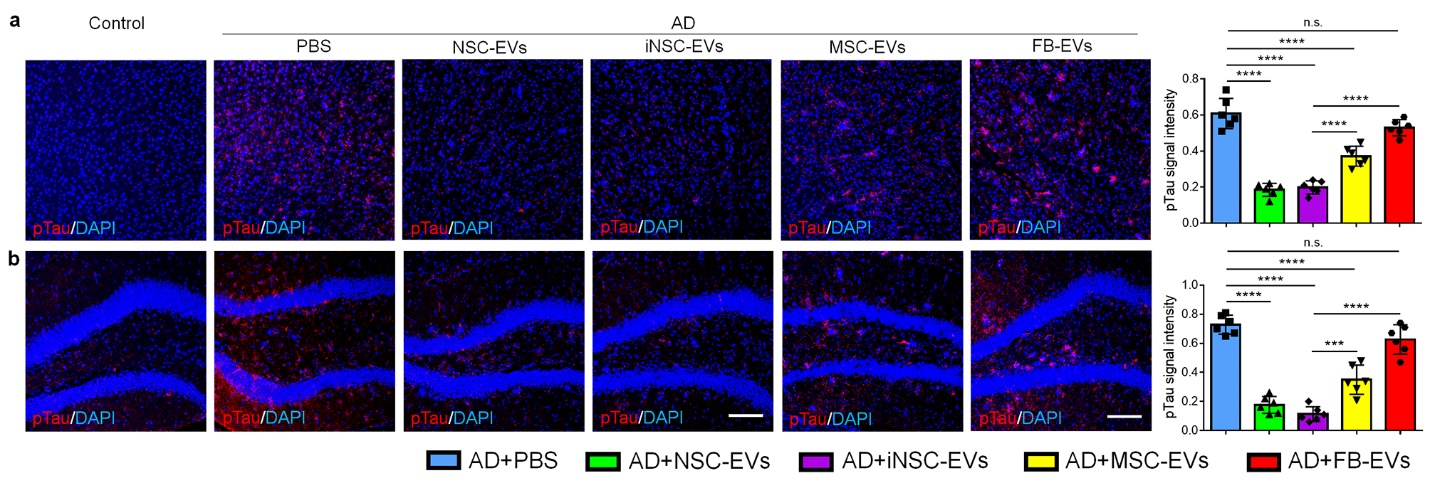


**Fig. S22.** **Intravenous administration of stem cell-derived EVs alleviates pTau propagation in the brains of 5×FAD mice.**

(**a**) Representative confocal microscopy images of pTau immunoreactivity in the PFC at ×20 magnification. The intensity of pTau signal in the PFC, quantified using ImageJ, was provided in the right panel (n = 6). (**b**) Representative confocal microscopy images of pTau immunoreactivity in the hippocampus at ×20 magnification. The intensity of pTau signal in the hippocampus, quantified using ImageJ, was provided in the right panel (n = 6). Scale bar: 200 μm. Error bars denote s.d.. *** and **** denote *p* < 0.001 and *p* < 0.0001, respectively. The statistical difference among groups was assessed with the parametric one-way ANOVA with post-hoc Bonferroni test.


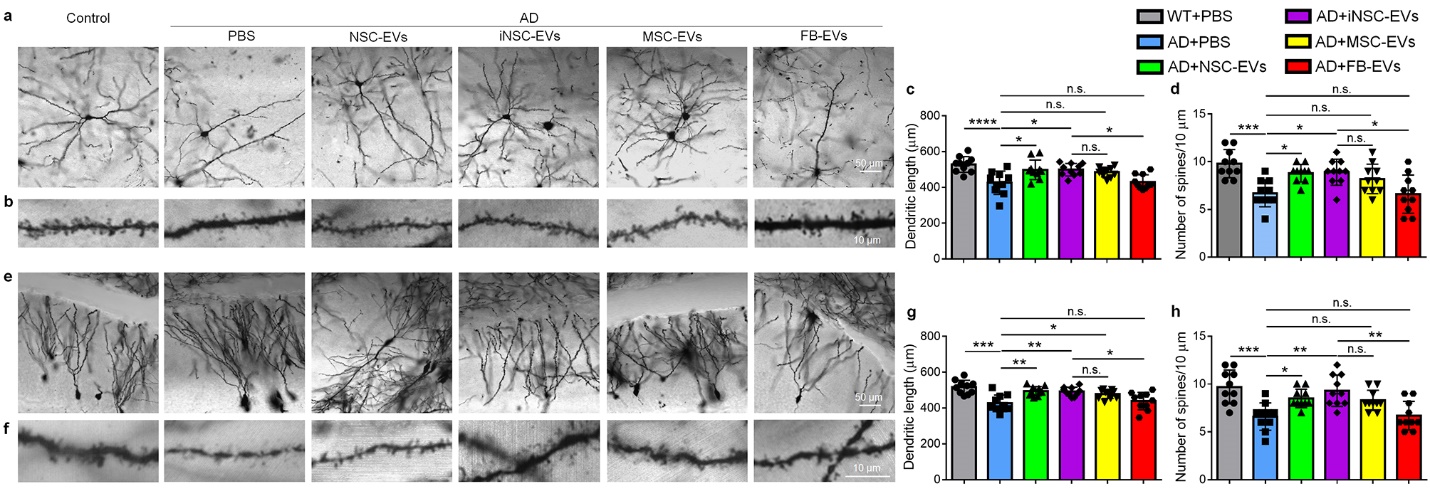


**Fig. S23.** **Intravenous administration of Stem cell-derived EVs restores dendritic length and spine density in the brains of 5×FAD mice.**

(**a**) Representative microscopy images of Golgi-Cox staining of cortical tissue samples at ×40 magnification. (**b**) Enlarged images demonstrating dendrites and spines. (**c**) Quantification of dendritic length of neurons in cortical tissue samples (n = 10). (**d**) Quantification of spine density in hippocampal tissue samples (n = 10). (**e**) Representative microscopy images of Golgi-Cox staining of hippocampal tissue samples at ×40 magnification. (**f**) Enlarged images demonstrating dendrites and spines. (**g**) Quantification of dendritic length of neurons in hippocampal tissue samples (n = 10). (**h**) Quantification of spine density in hippocampal tissue samples (n = 10). Error bars denote s.d.. *, **, ***, **** denotes *p* < 0.05, *p* < 0.01, *p* < 0.001, *p* < 0.0001, respectively. The statistical difference among groups was assessed with the parametric one-way ANOVA with post-hoc Bonferroni test.


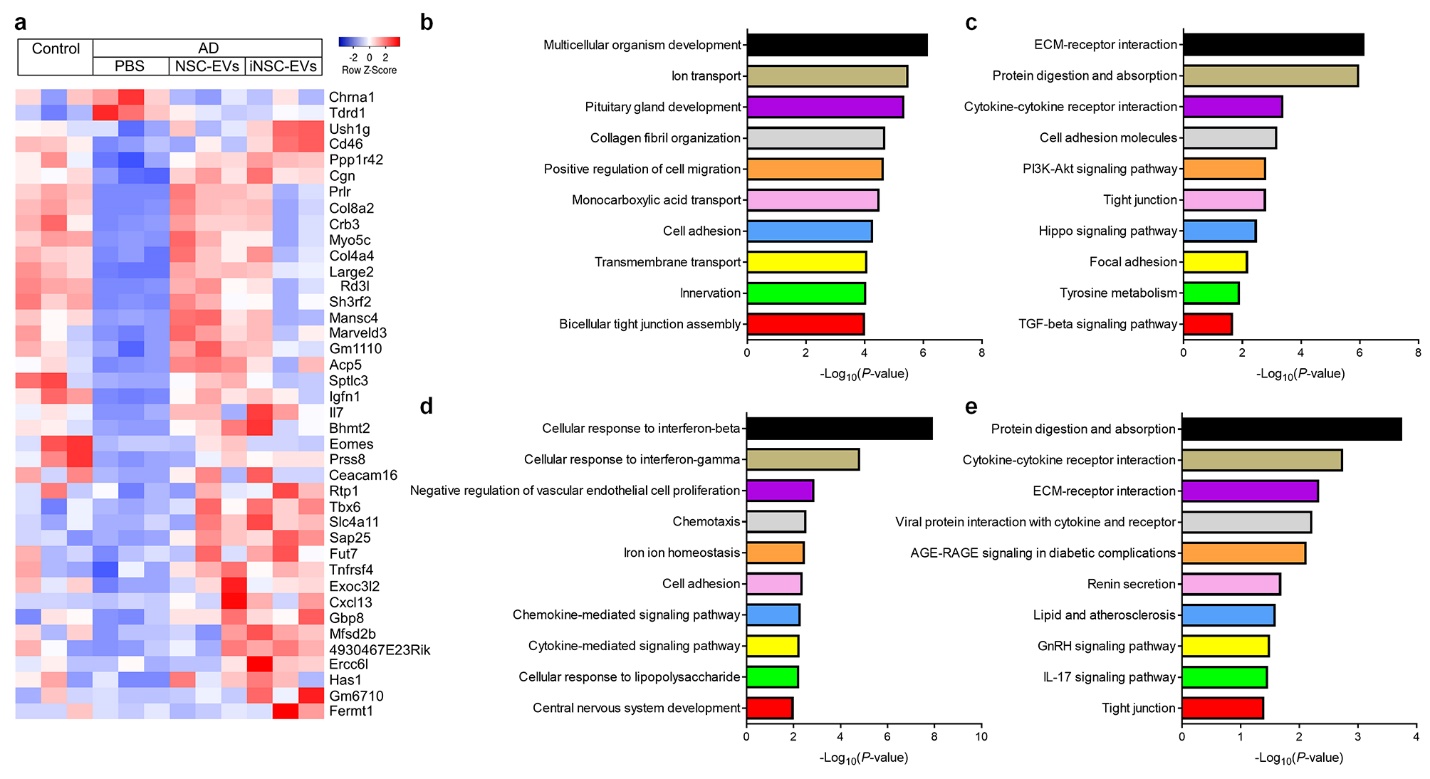


**Fig. S24.** **Intravenous administration of NSC- and iNSC-EVs alters gene expression profiles in 5×FAD mouse** **hippocampal tissues.**

(**a**) Heatmap of top 40 DEGs among groups. (**b**) The top 10 GO terms of DEGs in comparison between NSC-EV-inject mice and PBS controls. (**c**) The top 10 KEGG pathways of DEGs in comparison between NSC-EV-inject mice and PBS controls. (**d**) The top 10 GO terms of DEGs in comparison between iNSC-EV-inject mice and PBS controls. (**e**) The top 10 KEGG pathways of DEGs in comparison between iNSC-EV-inject mice and PBS controls.


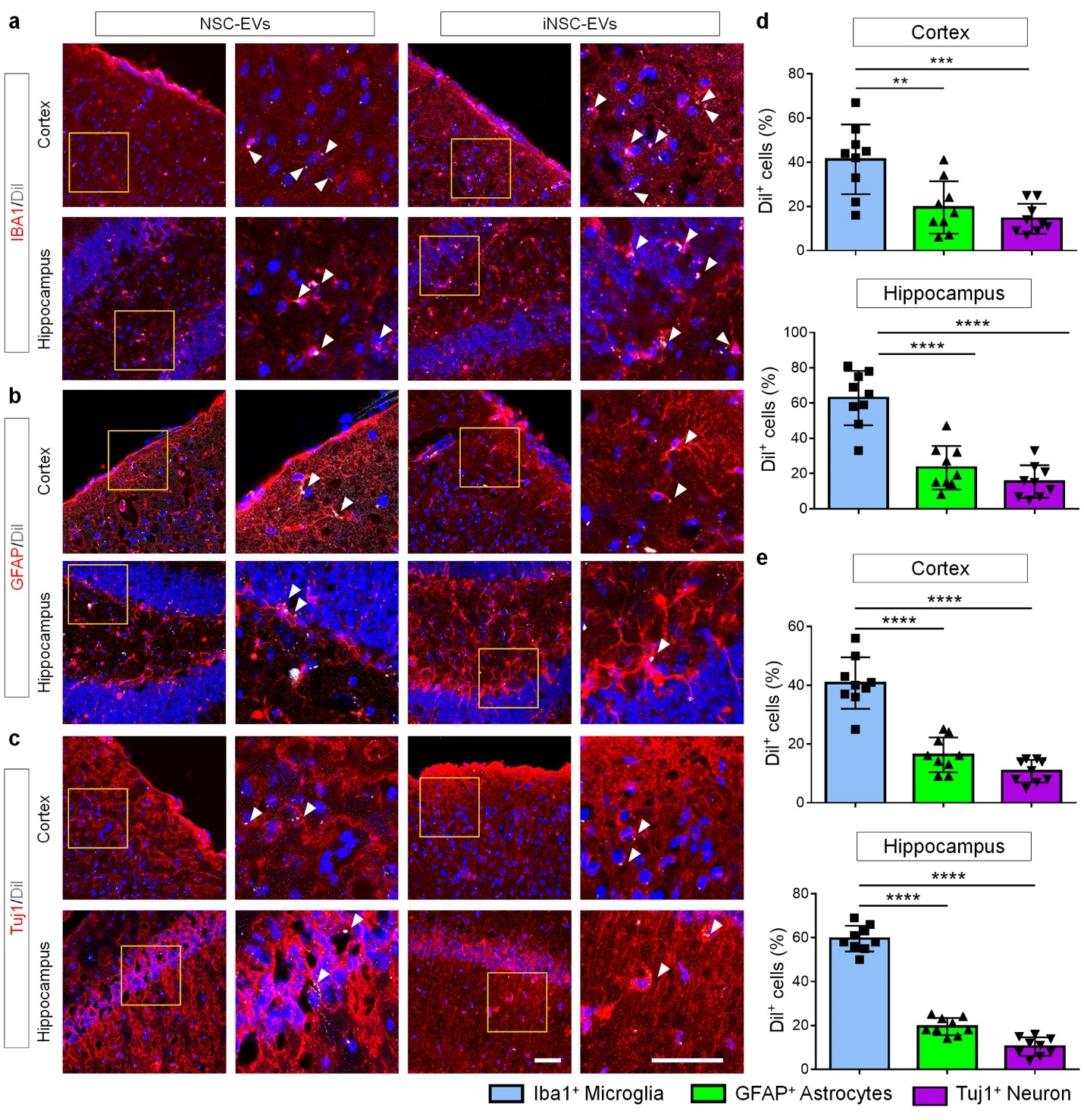


**Fig. S25. NSC- and iNSC-EVs are internalized mainly by microglia in the brain**.

Mice were injected intravenously with Dil-labeled EVs. Brain tissues were collected 5 min after EVs injection. (**a**) Representative confocal microscopy images of Iba1 and Dil immunoreactivities in the cortex and hippocampus. (**b**) Representative confocal microscopy images of GFAP and Dil immunoreactivities in the cortex and hippocampus. (**c**) Representative confocal microscopy images of Tuj1 and Dil immunoreactivities in the cortex and hippocampus. (**d**) Quantitation analysis for the comparison of NSC-EVs intake in microglia, astrocytes, and neurons in the PFC and hippocampus. (**e**) Quantitation analysis for the comparison of iNSC-EVs intake in microglia, astrocytes, and neurons in the PFC and hippocampus. Images at the right panels were high-magnification images of the corresponding small box area from the left panels in each group. Arrows indicate overlapping signals. Scale bar: 50 μm. Error bars denote s.d.. **, ***, and **** denote *p* < 0.01, *p* < 0.001, and *p* < 0.0001, respectively. The statistical difference among groups was assessed with the parametric one-way ANOVA with post-hoc Bonferroni test.


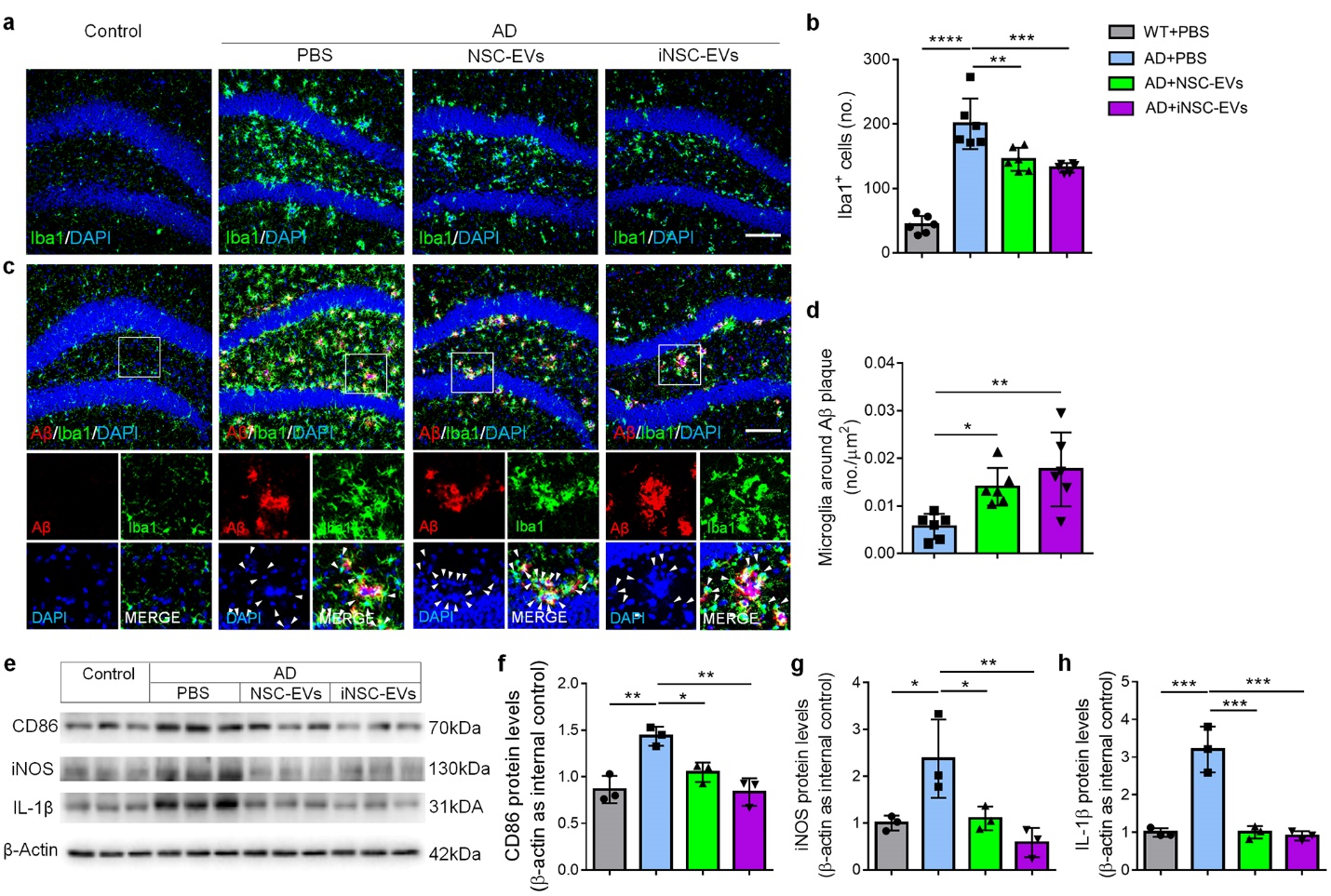


**Fig. S26.** **Intravenous administration of NSC-EVs and iNSC-EVs inhibits microglial activation and enhances microglial Aβ responses in the hippocampi of 5×FAD mice.**

(**a**) Representative confocal microscopy images of Iba1 immunoreactivity in the hippocampus at ×20 magnification. Numbers of immunoreactive cells in each group were given on the right panel (n = 6). (**b**) Representative confocal microscopy images of Iba1 and Aβ immunoreactivity in the hippocampus at ×20 magnification. Numbers of Iba1^+^ cells around the surface of Aβ plaques were given on the right panel (n = 6). (**c**) Representative blot (left) and quantification (right) of CD86, iNOS, and IL-1β protein expression levels in the hippocampus (n = 3). Western blotting data were normalized to β-actin. Scale bar: 200 μm. Error bars denote s.d.. *, **, ***, and **** denote *p* < 0.05, *p* < 0.01, *p* < 0.001, and *p* < 0.0001, respectively. The statistical difference among groups was assessed with the parametric one-way ANOVA with post-hoc Bonferroni test.


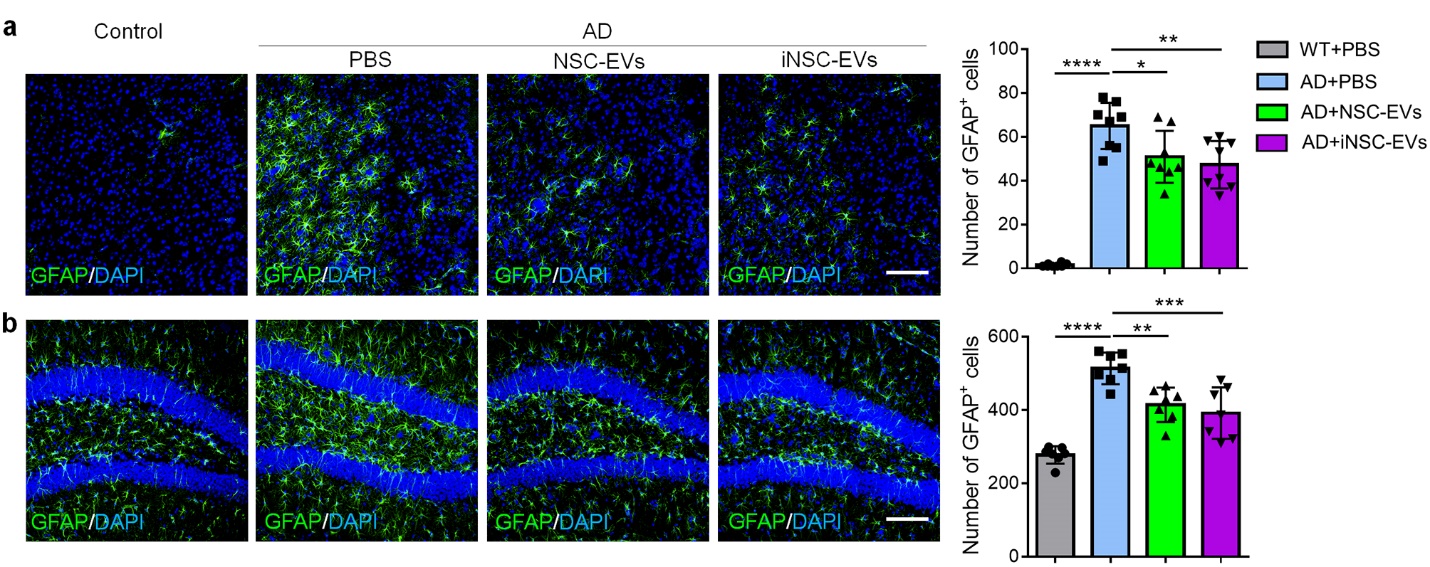


**Fig. S27.** **Intravenous administration of NSC- and iNSC-EVs inhibits astrocyte activation in 5×FAD mouse brains.**

(**a**) Representative confocal microscopy images of GFAP immunoreactivity in the prefrontal cortex at ×20 magnification. Numbers of immunoreactive cells in each group were given on the right panel (n = 8). (**b**) Representative confocal microscopy images of GFAP immunoreactivity in the hippocampus at ×20 magnification. Numbers of immunoreactive cells in each group were given on the right panel (n = 7). Scale bar: 200 μm. Error bars denote s.d.. *, **, **** denote *p* < 0.05, *p* < 0.01, and *p* < 0.0001, respectively. The statistical difference among groups was assessed with the parametric one-way ANOVA with post-hoc Bonferroni test.


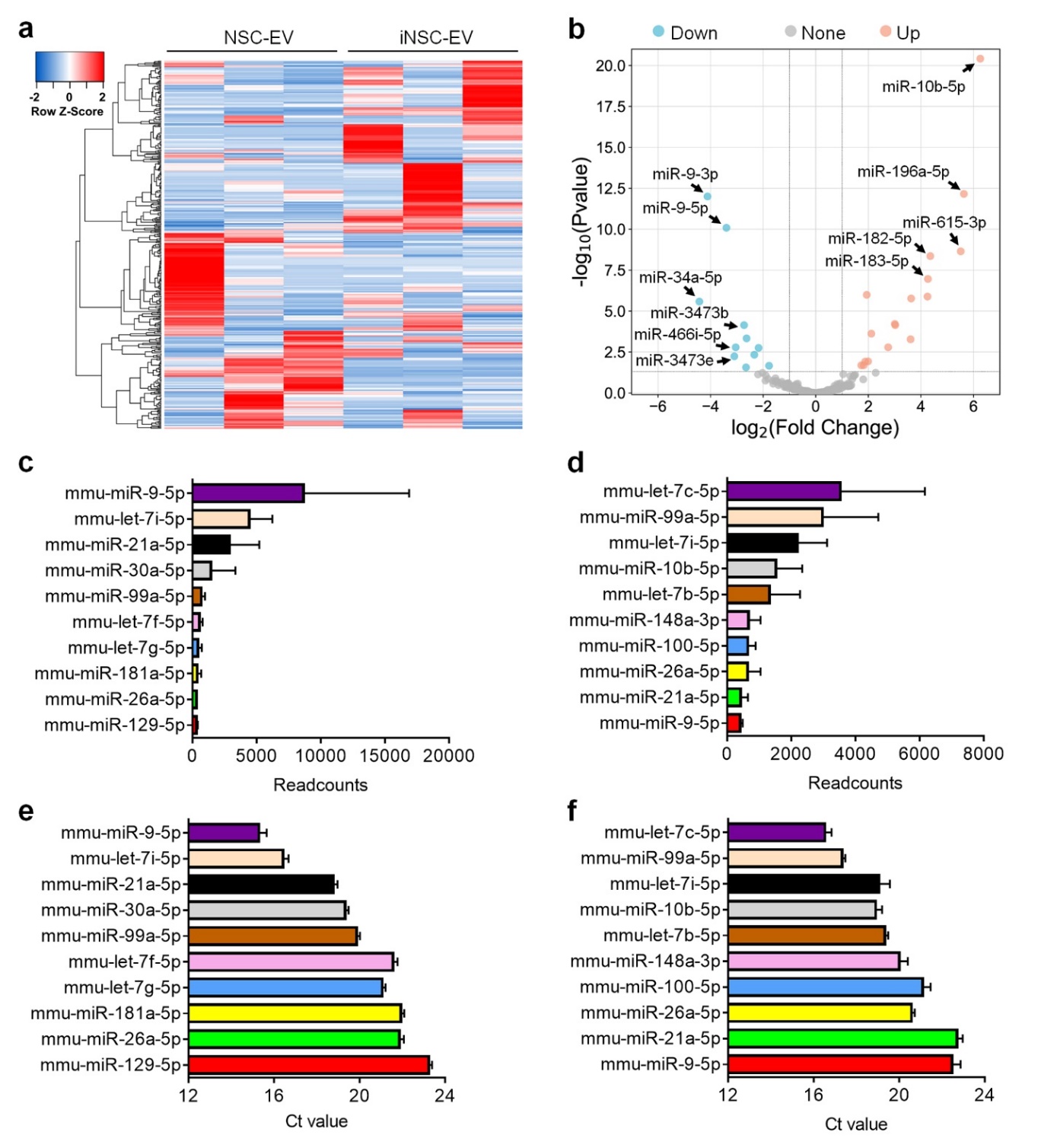


**Fig. S28.** **Intravenous administration of NSC- and iNSC-EVs alters gene expression profiles in 5×FAD mouse hippocampal tissues.**

(**a**) Heatmap of all detected miRNAs in NSC-EVs and iNSC-EVs by microarray. (**b**) Volcano plot of all detected miRNAs in NSC-EVs and iNSC-EVs. (**c**) The top 10 miRNAs with highest readcounts in NSC-EVs. (**d**) The top 10 miRNAs with highest readcounts in iNSC-EVs. (**e**) The qRT-PCR validation of the top 10 miRNAs with highest readcounts in NSC-EVs. (**f**) The qRT-PCR validation of the top 10 miRNAs with highest readcounts in iNSC-EVs.


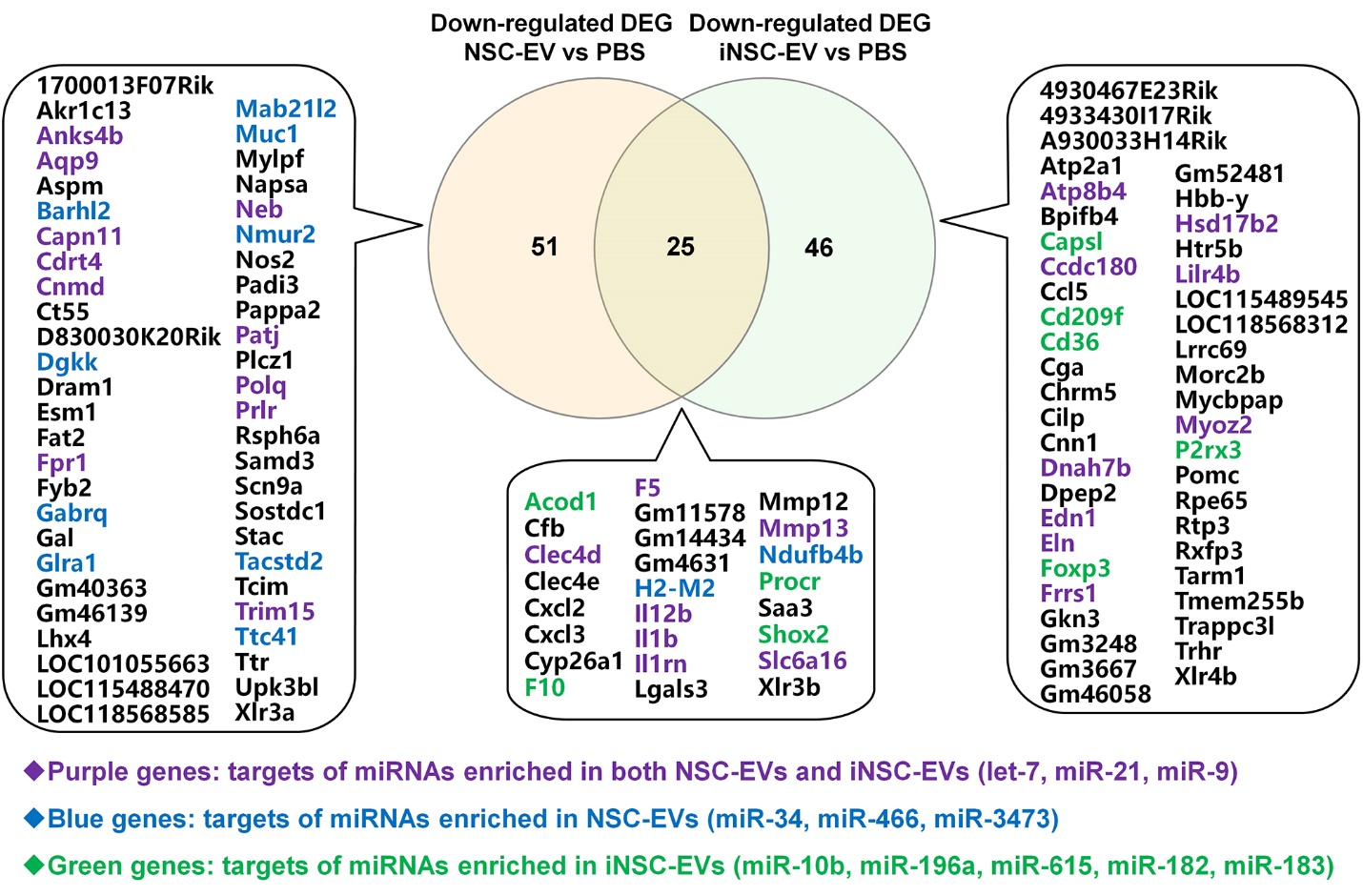


**Fig. S29.** **Intravenous administration of NSC- and iNSC-EVs alters gene expression profiles in 5×FAD mouse hippocampal tissues.**

(**a**) Heatmap of top 40 DEGs among groups. (**b**) The top 10 GO terms of DEGs in comparison between NSC-EV-inject mice and PBS controls. (**c**) The top 10 KEGG pathways of DEGs in comparison between NSC-EV-inject mice and PBS controls. (**d**) The top 10 GO terms of DEGs in comparison between iNSC-EV-inject mice and PBS controls. (**e**) The top 10 KEGG pathways of DEGs in comparison between iNSC-EV-inject mice and PBS controls.


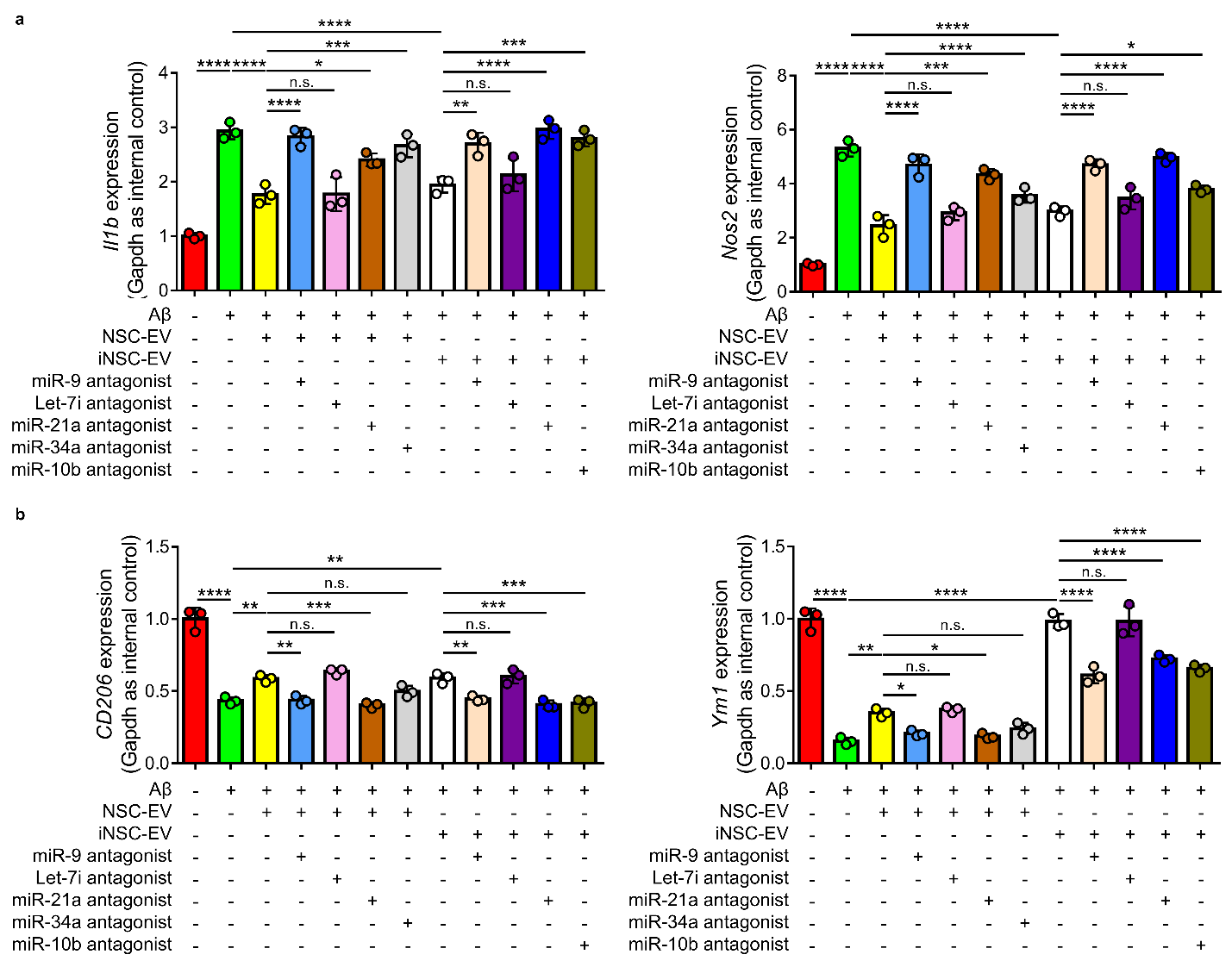


**Fig. S30.** **Intravenous administration of NSC- and iNSC-EVs alters gene expression profiles in 5×FAD mouse hippocampal tissues.**

Primary mouse microglia were stimulated by Aβ_1-42_, followed by EV treatment and the transfection of either miR-9, let-7i, miR-21a, miR-34a, or miR-10b antagonists for 2 days. (**a**) The transcript levels of pro-inflammatory genes *Il1b* and *Nos2* in microglia were determined by qRT-PCR. (**b**) The transcript levels of anti-inflammatory genes *CD206* and *Ym1* in microglia were determined by qRT-PCR. Error bars denote s.d.. n.s. denotes no significance. *, **, ***, and **** denote *p* < 0.05, *p* < 0.01, *p* < 0.001, and *p* < 0.0001, respectively. The statistical difference among groups was assessed with the parametric one-way ANOVA with post-hoc Bonferroni test.


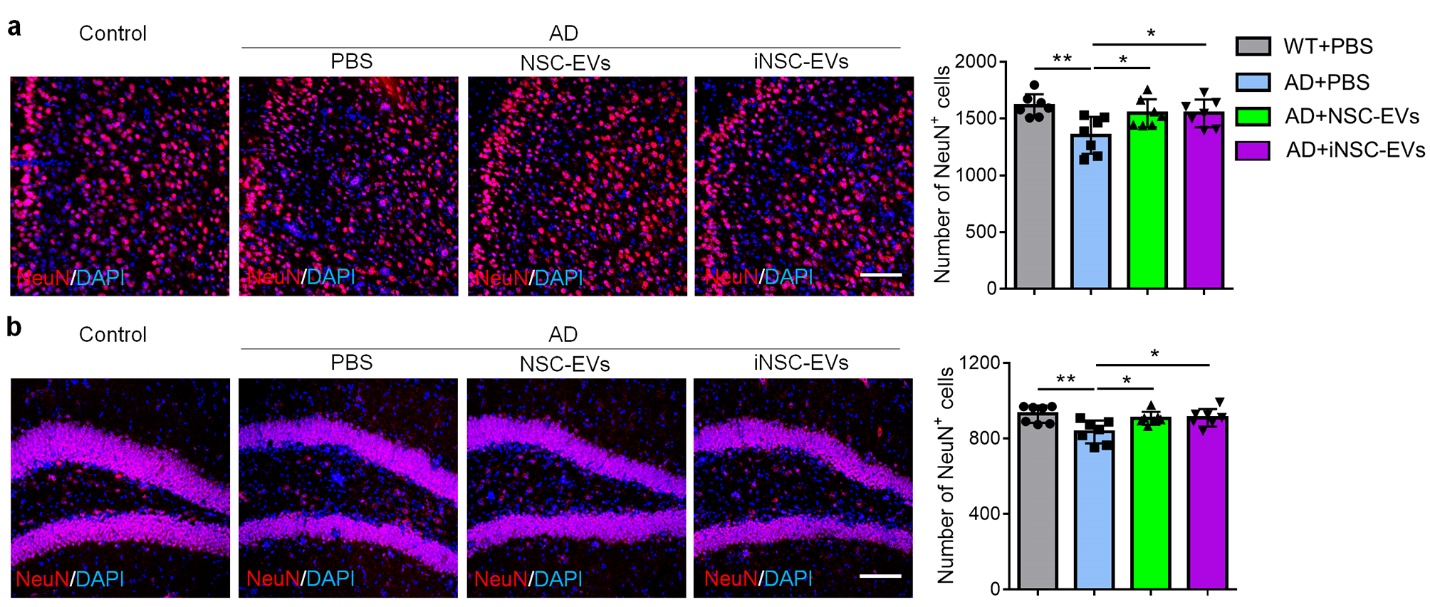


**Fig. S31. Intravenous administration of NSC- and iNSC-EVs rescues neuronal loss in 5×FAD mouse brains**.

(**a**) Representative confocal microscopy images of NeuN immunoreactivity in the prefrontal cortex at ×20 magnification. Numbers of NeuN^+^ cells in each group were given on the right panel. (**b**) Representative confocal microscopy images of NeuN immunoreactivity in the hippocampus at ×20 magnification. Numbers of NeuN^+^ cells in each group were given on the right panel. N = 4. Scale bar: 200 μm. Error bars denote s.d.. * and ** denote *p* < 0.05 and *p* < 0.01, respectively. The statistical difference among groups was assessed with the parametric one-way ANOVA with post-hoc Bonferroni test.


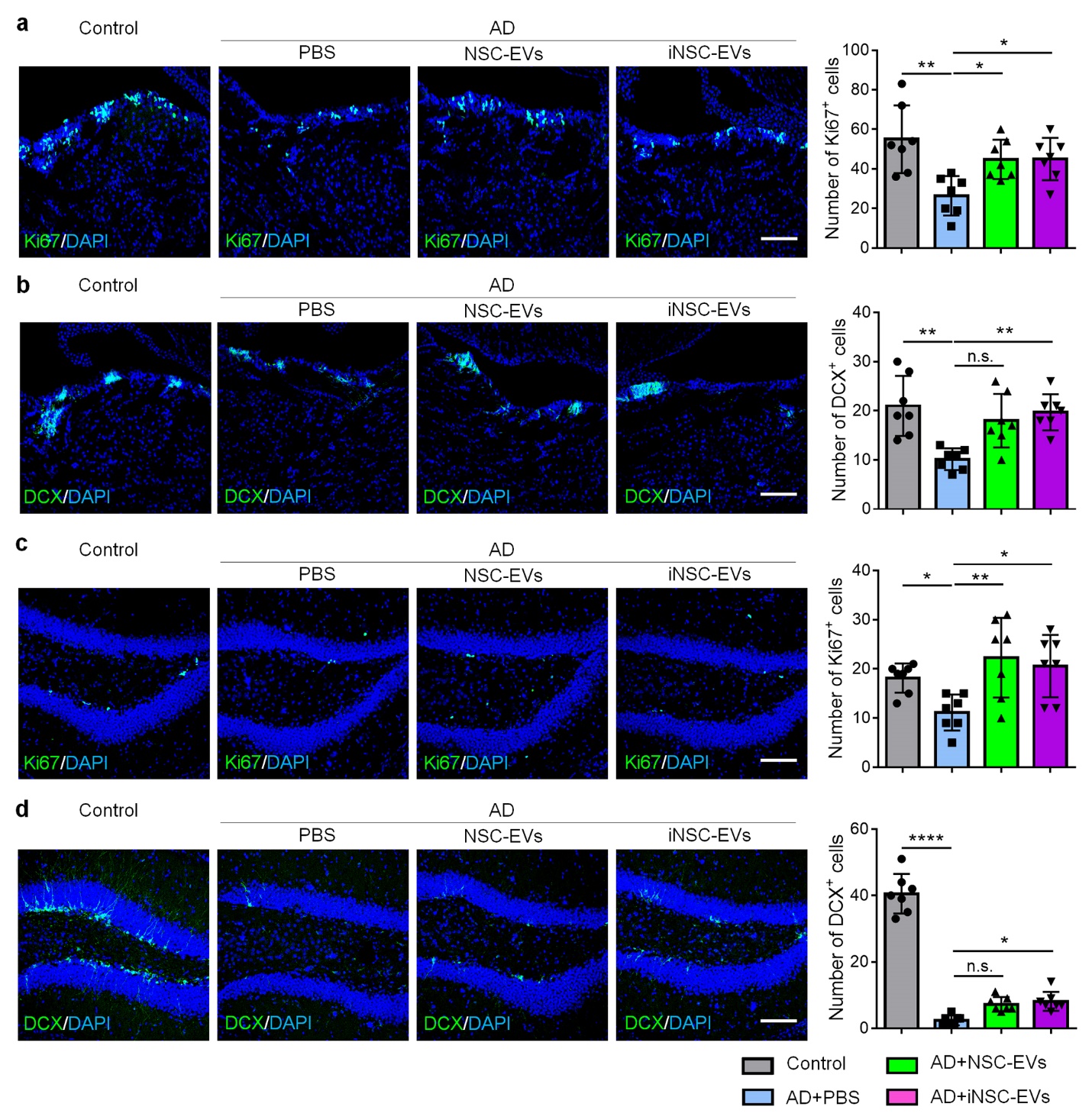


**Fig. S32. Intravenous administration of NSC- and iNSC-EVs promotes neuroregeneration in 5×FAD mouse brains.**

(**a, b**) Representative confocal microscopy images of Ki67 (**a**) and DCX (**b**) immunoreactivity in the SVZ at ×20 magnification. Numbers of immunoreactive cells in each group were given on the right panel. (**c, d**) Representative confocal microscopy images of Ki67 (**c**) and DCX (**d**) immunoreactivity in the hippocampus at ×20 magnification. Numbers of immunoreactive cells in each group were given on the right panel. N = 4. Scale bar: 200 μm. Error bars denote s.d.. n.s. denotes no significance. *, **, **** denote *p* < 0.05, *p* < 0.01, and *p* < 0.0001, respectively. The statistical difference among groups was assessed with the parametric one-way ANOVA with post-hoc Bonferroni test.
